# Evaluation of an audit and feedback intervention to reduce gentamicin prescription errors in newborn treatment (ReGENT) in neonatal inpatient care in Kenya: a controlled interrupted time series study protocol

**DOI:** 10.1186/s13012-022-01203-w

**Published:** 2022-05-16

**Authors:** Timothy Tuti, Jalemba Aluvaala, Lucas Malla, Grace Irimu, George Mbevi, John Wainaina, Livingstone Mumelo, Kefa Wairoto, Dolphine Mochache, Christiane Hagel, Michuki Maina, Mike English, on behalf of The Clinical Information Network Group, Juma Vitalis, Nyumbile Bonface, Roselyne Malangachi, Christine Manyasi, Catherine Mutinda, David Kibiwott Kimutai, Rukia Aden, Caren Emadau, Elizabeth Atieno Jowi, Cecilia Muithya, Charles Nzioki, Supa Tunje, Penina Musyoka, Wagura Mwangi, Agnes Mithamo, Magdalene Kuria, Esther Njiru, Mwangi Ngina, Penina Mwangi, Rachel Inginia, Melab Musabi, Emma Namulala, Grace Ochieng, Lydia Thuranira, Felicitas Makokha, Josephine Ojigo, Beth Maina, Mary Waiyego, Bernadette Lusweti, Angeline Ithondeka, Julie Barasa, Meshack Liru, Elizabeth Kibaru, Alice Nkirote Nyaribari, Joyce Akuka, Joyce Wangari, Amilia Ngoda, Aggrey Nzavaye Emenwa, Patricia Nafula Wesakania, George Lipesa, Jane Mbungu, Marystella Mutenyo, Joyce Mbogho, Joan Baswetty, Ann Jambi, Josephine Aritho, Beatrice Njambi, Felisters Mucheke, Zainab Kioni, Jeniffer, Lucy Kinyua, Margaret Kethi, Alice Oguda, Salome Nashimiyu Situma, Nancy Gachaja, Loise N. Mwangi, Ruth Mwai, Irginia Wangari Muruga, Nancy Mburu, Celestine Muteshi, Abigael Bwire, Salome Okisa Muyale, Naomi Situma, Faith Mueni, Hellen Mwaura, Rosemary Mututa, Caroline Lavu, Joyce Oketch, Jane Hore Olum, Orina Nyakina, Faith Njeru, Rebecca Chelimo, Margaret Wanjiku Mwaura, Ann Wambugu, Epharus Njeri Mburu, Linda Awino Tindi, Jane Akumu, Ruth Otieno, Slessor Osok, Seline Kulubi, Susan Wanjala, Pauline Njeru, Rebbecca Mukami Mbogo, John Ollongo, Samuel Soita, Judith Mirenja, Mary Nguri, Margaret Waweru, Mary Akoth Oruko, Jeska Kuya, Caroline Muthuri, Esther Muthiani, Esther Mwangi, Joseph Nganga, Benjamin Tanui, Alfred Wanjau, Judith Onsongo, Peter Muigai, Arnest Namayi, Elizabeth Kosiom, Dorcas Cherop, Faith Marete, Johanness Simiyu, Collince Danga, Arthur Otieno Oyugi, Fredrick Keya Okoth

**Affiliations:** 1 KEMRI-Wellcome Trust Research Programme, Nairobi, Kenya; 2 Department of Paediatrics and Child Health, University of Nairobi, Nairobi, Kenya; 3 London School of Hygiene and Tropical Medicine, London, UK; 4 Nuffield Department of Medicine, University of Oxford, Oxford, UK

**Keywords:** Audit and feedback, Clinical guidelines, Newborns, Inappropriate prescribing, Low- and middle-income settings

## Abstract

**Background:**

Medication errors are likely common in low- and middle-income countries (LMICs). In neonatal hospital care where the population with severe illness has a high mortality rate, around 14.9% of drug prescriptions have errors in LMICs settings. However, there is scant research on interventions to improve medication safety to mitigate such errors. Our objective is to improve routine neonatal care particularly focusing on effective prescribing practices with the aim of achieving reduced gentamicin medication errors.

**Methods:**

We propose to conduct an audit and feedback (A&F) study over 12 months in 20 hospitals with 12 months of baseline data. The medical and nursing leaders on their newborn units had been organised into a network that facilitates evaluating intervention approaches for improving quality of neonatal care in these hospitals and are receiving basic feedback generated from the baseline data. In this study, the network will (1) be expanded to include all hospital pharmacists, (2) include a pharmacist-only professional WhatsApp discussion group for discussing prescription practices, and (3) support all hospitals to facilitate pharmacist-led *continuous medical education* seminars on prescription practices at hospital level, i.e. default intervention package. A subset of these hospitals (*n* = 10) will additionally (1) have an additional hospital-specific WhatsApp group for the pharmacists to discuss local performance with their local clinical team, (2) receive detailed A&F prescription error reports delivered through mobile-based dashboard, and (3) receive a PDF infographic summarising prescribing performance circulated to the clinicians through the hospital-specific WhatsApp group, i.e. an extended package.

Using interrupted time series analysis modelling changes in prescribing errors over time, coupled with process fidelity evaluation, and WhatsApp sentiment analysis, we will evaluate the success with which the A&F interventions are delivered, received, and acted upon to reduce prescribing error while exploring the extended package’s success/failure relative to the default intervention package.

**Discussion:**

If effective, these theory-informed A&F strategies that carefully consider the challenges of LMICs settings will support the improvement of medication prescribing practices with the insights gained adapted for other clinical behavioural targets of a similar nature.

**Trial registration:**

PACTR, PACTR202203869312307. Registered 17th March 2022.

**Supplementary Information:**

The online version contains supplementary material available at 10.1186/s13012-022-01203-w.

Contributions to the literature

- This study is one of the first in a low- and middle-income country (LMIC) to evaluate at the clinical team level a comprehensive healthcare-specific feedback theory used to design and implement feedback to improve medication prescribing accuracy during inpatient neonatal care.
- Findings will advance our knowledge about how clinical care teams utilising different approaches to feedback strategies work to best improve prescribing practices in neonatal care in LMICs.
- Such evidence will advance our knowledge on how to develop scalable and effective medication safety quality improvement approaches and improve health workers’ motivation to focus on treatment guidelines adherence.

## Introduction

Improving medication safety is a global priority as medication errors arising from prescribing, dispensing, transcribing, administering, and monitoring medicines can cause severe harm and increase healthcare costs [1–3]. Most evidence on medication safety in routine healthcare settings is from high-income countries (HICs) [3]. From the limited findings available, medication errors might be substantively higher in low- and middle-income countries (LMICs) [4–6], especially in neonatal (i.e. first 28 days of life) hospital care [7, 8] where the population with severe illness has high mortality [9]. While around 14.9% of drug prescriptions have errors in neonatal care settings [7], there is scant research on interventions to improve medication safety to mitigate such errors in LMICs.

Electronic prescribing (i.e. e-Prescribing with in-built error checking) might improve neonatal care medication practices, but may not be feasible to implement in many public hospitals in LMICs due to resource constraints and level of maturity of electronic health records (EHRs) [10, 11]. Quality improvement (QI) programmes have had some success in improving clinical outcomes (i.e. 35–50% reduction in prescription errors in neonatal care), might be more feasible in many LMIC settings, and could benefit from context-appropriate audit and feedback (A&F) strategies and cycles [12–15].

Audit requires data. It may come from (1) intermittent record audit, (2) digital data on patients and prescribing, or (3) EHRs and e-prescribing which require low, moderate, and high technological capacities, respectively. Feedback is posited to reduce unsafe prescribing practices especially when it has multiple components (e.g. education), involves key agents (facilitators and champions) such as pharmacists, or addresses individual and team goals [16, 17]. The roles of key agents (facilitators and champions), for example pharmacists, for prescribing practices improvement have only rarely been empirically tested [18, 19]. As we have observed in Kenyan hospital practice, there is little interaction between clinical teams and pharmacists to guide medication prescribing with medications reconstituted and administered by nurses on the ward (e.g. gentamicin); pharmacists typically only get involved for inpatient care when potentially toxic medications (e.g. chemotherapy) are administered, but this is a rare event confined to higher level hospitals. This context provides an opportunity to evaluate including pharmacists as key agents in improving prescribing practices in neonatal care.

Additionally, sources of data are limited in many LMICs on different modes of feedback to individuals or teams. Feedback directly to clinicians on their performance is posited to be most effective [20], However, healthcare workers (HCWs) are few in number in many LMICs with a finite supply of time and resources to engage with feedback [20]. The design choices in LMICs A&F studies should take account of both the specific characteristics of these contexts and existing knowledge and theory [21, 22]. To be useful, studies need to consider external validity, whether the data or technologies needed to support A&F approaches might be available at scale, what advantages might be gained by leveraging local clinical champions, and the value and practicality of feedback at team or individual levels. Their design also needs to consider that the effect of A&F interventions may diminish over time so they should aim to go beyond a single feedback cycle or short-term intervention period [23] and, ideally, should move beyond simple before-after designs.

In LMIC, neonatal infections are one of the most common causes of death during the neonatal period [24], and the increase in global prevalence of antibiotic resistance in neonatal units indicates a need for improved antimicrobial stewardship [25]. Evidence from A&F interventions suitable for the Kenyan context could advance this agenda as well as improving patient safety. We focus on prescription accuracy for Gentamicin since:

- Gentamicin is on the World Health Organization (WHO) essential medicines list [26] and is the first-line drug for treatment for neonatal sepsis in newborns in Kenya and LMICs and is even being used for community-based treatments [25–28].
- Gentamicin is associated with well-known risks of toxicity if doses are too high for too long [29], while if doses are too low, it is less effective in bacterial killing. Inadequate dosing is therefore important because of the increase in antimicrobial resistance globally [25, 27, 28].
- WHO and Kenyan dosage guidelines are based on weight and post-natal age, and so, prescribing is slightly more complicated than other drugs increasing the risk of prescribing errors [30, 31].
- We already know that approximately 14% of the gentamicin prescription provided in the Kenyan hospitals we work with has prescribing errors, i.e. aggregate of dosages that are either too high or too low (which we explain in detail in the “Study setting” sub-section of the “Design and methodology” section), and even higher rates have been reported previously from Kenya [32, 33].

Gentamicin prescribing therefore presents a good case to study whose findings could be applied to other medications prescribed in Kenya and other settings. Penicillin is typically prescribed together with gentamicin for neonatal sepsis in Kenya and elsewhere in line with national and global guidance. It is much less likely to cause patient harm if there are prescription errors and has a much lower prescription error rate in our setting (unpublished data, further detail provided in the “Study setting” sub-section of the “Design and methodology”). For this reason, we focus on gentamicin as a priority in terms of need to intervene [8, 29, 34].

Key particularities about LMICs study settings like Kenya that make them different from HIC studies and are important considerations for A&F intervention design for reducing such prescribing errors include the following:

1. The lack of electronic prescribing for inpatients, thus, no automated dosage checks or decision support is available in these sites [35].
2. Junior clinical personnel do 80% of the admitting/prescribing and rotate thrice monthly [36] and, until recently, tend to have had limited neonatal training.
3. There are only one or two and sometimes no paediatricians for these hospitals [37], so the junior prescribers often have limited supervision, for example on ward rounds, from any specialist.
4. The pharmacists are also very few and, in most places, play no direct role in ward-based oversight and education of prescribers on newborn units (NBUs) [31, 38].
5. There are national guidelines that are widely disseminated to clinicians and are approximately the same as the WHO guidance that should govern prescription practices [27].
6. Routine therapeutic monitoring of gentamicin or other aminoglycoside drug levels is not available in any site.
7. Empiric antibiotic treatment is very common with clinicians very rarely having access to diagnostics for sepsis such as blood cultures.

More specifically, we work with 20 first-referral level hospitals organised into a Clinical Information Network (CIN) [11, 13, 39] where the hospitals receive 3-monthly clinical A&F reports on the quality of care they provide for common conditions, which include a summary of prescription error rates for gentamicin [30]. More details are provided later on in the “Study setting” sub-section of the “Design and methodology” section. In this study, we will use a pharmacist-facilitated A&F intervention that is guided by the Clinical Performance Feedback Intervention Theory (CP-FIT) and builds on the principles of previously reported studies [16, 20, 40], in which clinical pharmacists are conceptualised as QI champions, and we anticipate they will work with doctors and nurses in their hospital’s neonatal units to act upon feedback (Table 1).

**Table 1:** Primer on Clinical Performance Feedback Intervention Theory (CP-FIT)

**Fig. 1:**
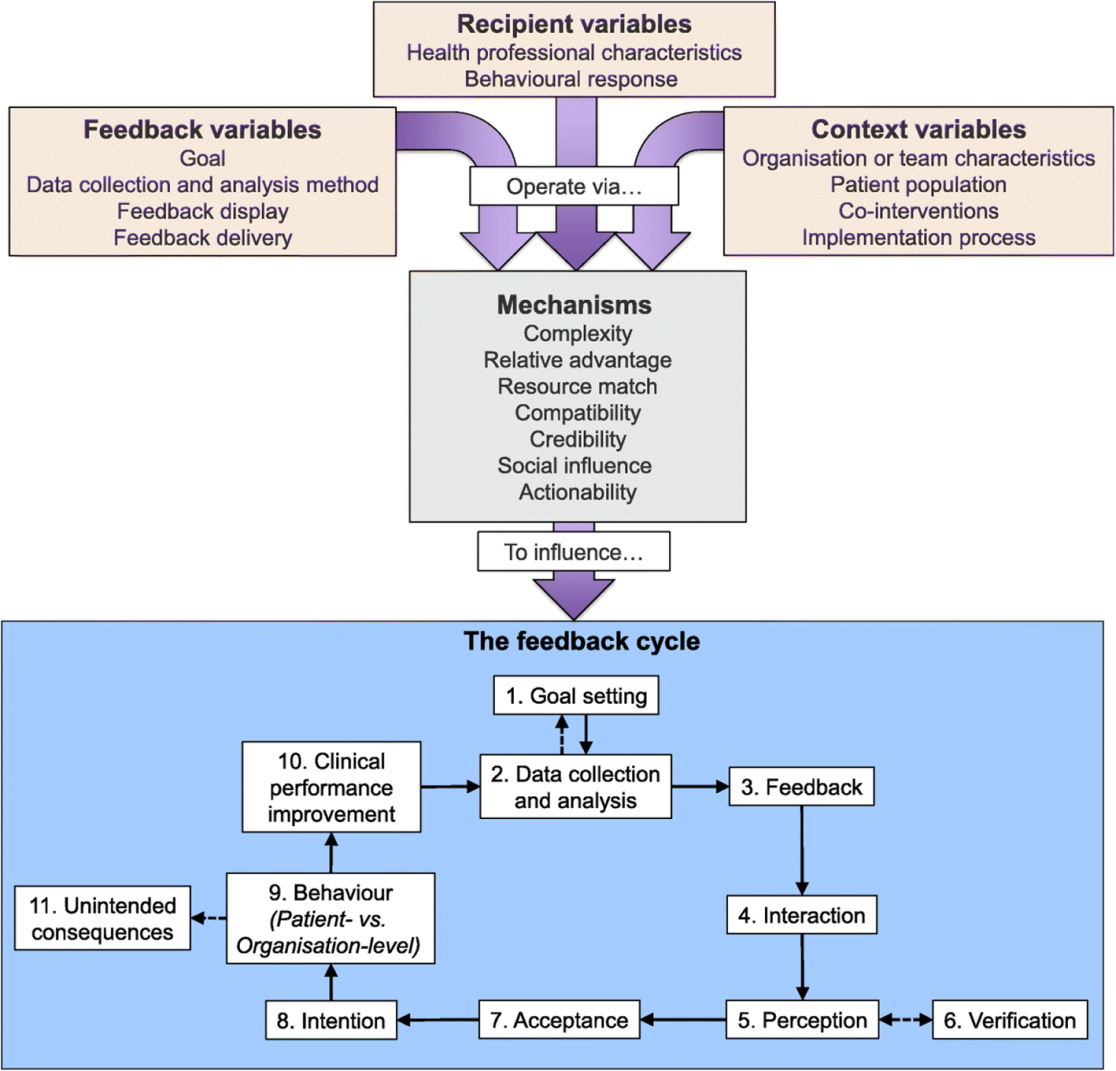
Clinical Performance Feedback Intervention Theory’s variables and explanatory mechanisms and their influence on the feedback cycle. Solid arrows are necessary pathways for successful feedback. Dotted arrows represent potential pathways

CP-FIT is synthesised from 65 qualitative studies of 73 A&F interventions and 30 pre-existing theories and describes causal pathways of feedback [20]. It states that effective feedback is a cyclical process of *goal setting*, *data collection and analysis*, *feedback*, recipient *interaction*, *perception*, and *acceptance* of the feedback, followed by *intention*, *behaviour*, and *clinical performance improvement* (the feedback cycle) (Fig. 1) [20]. Feedback becomes less effective if any individual process fails causing progress round the feedback cycle to stop and is influenced by variables relating to the feedback itself (its *goal*, *data collection and analysis methods*, *feedback display*, and *feedback delivery*), the recipient (*health professional characteristics* and *behavioural response*), and context (*organisation or team characteristics*, *patient population*, *co-interventions,* and *implementation process*) (Fig. 1) [20]. These variables exert their effects via explanatory mechanisms of *complexity*, *relative advantage*, *resource match*, *compatibility*, *credibility*, *social influence*, and *actionability* and are summarised by three propositions [20]: (a) Capacity limitations: Healthcare professionals and organisations have a finite capacity to engage with and respond to feedback; interventions that require less work, supply, additional resource, or are considered worthwhile enough to justify investment are most effective. (b) Identity and culture: Healthcare professionals and organisations have strong beliefs regarding how patient care should be provided that influence their interactions with feedback; those that align with and enhance these aspects are most effective. (c) Behavioural induction: Feedback interventions that successfully and directly support clinical behaviours for individual patients are most effective.	

We will explicitly target feedback variables that are theorised to make feedback effective. These include the following: (1) an important clinical goal targeted by the feedback intervention, (2) using verifiable data collection and analysis methods that enhance accuracy, credibility, and acceptance of feedback, (3) employing understandable feedback displays that reinforce positive healthcare workers (HCWs) intentions and behaviours, and (4) employing feedback that targets HCWs teams’ inherent motivation to improve an important practice (Fig. 1) [20]. To enhance feedback in some of these thematic areas and in some sites, we will use a novel electronic, interactive mobile-friendly dashboard that provides summaries of prescribing performance auto-updated monthly. The inclusion of pharmacists as key agents of A&F design and implementation is informed by CP-FIT theory [20], with the expectation that they serve a fundamental role of offering clinical leadership in prescribing practices [18, 19].

High-quality contemporaneously collected data on drug prescribing is needed to provide timely A&F. Investment in such systems is justified at scale if they result in better clinical practices, especially in sub-Saharan Africa (SSA)’s public healthcare systems [22, 41]. Therefore, it is important to assess the advantages of such approaches against more basic improvement or A&F strategies with fewer data demands. Conducting head-to-head experiments informed by current empirical and theoretical insights in A&F research is also a global priority [21, 41]. Head-to-head comparisons offer additional advantages. They allow more direct examination of the fidelity of delivery of different approaches, mechanisms of action, potential confounders, and effect modifiers of A&F implementation strategies. They go beyond causal description to help interpret and determine the generalisability of evaluation findings to produce transferable learning [21, 22]. When coupled with data collected over longer time periods, they can help evaluate any decay in effects of interventions [21].

Therefore, this study will use interrupted time series (ITS) comparison of gentamicin error rates from pre- and post-A&F intervention periods to compare enhanced A&F interventions to the usual routine feedback reports that the CIN hospitals receive (presented in documents sent to hospitals); the evaluation of the relative effect of the enhanced A&F intervention over the basic feedback reports will also include comparison of two versions of enhanced A&F intervention packages including an assessment of the implementation process of both. Effectively, the null hypotheses are that over time, (1) there is no difference in gentamicin prescribing error rates in hospitals receiving enhanced A&F compared with when they have basic A&F interventions, and any time-based changes are due to secular performance trends, and (2) there are no differences in gentamicin prescribing errors between two sets of hospitals receiving two different forms of enhanced feedback.

Given that the primary outcome of this study being measured is the proportion of patients in newborn units receiving an inaccurate gentamicin prescription over time (i.e. incidence rate ratio), our objectives are threefold:

1. To evaluate if enhancing A&F intervention approaches over and above existing use of feedback reports reduces the prevalence of gentamicin prescribing errors (measured as an incidence rate ratio) in neonatal inpatient hospital care over time
2. To evaluate if an A&F package incorporating more Clinical Performance Feedback Intervention Theory (CP-FIT) components is more effective in reducing gentamicin prescribing errors in inpatient neonatal care compared to an A&F package incorporating fewer CP-FIT informed components (which is likely to be easier to scale across facilities)
3. To explore the value of the CP-FIT model as a guiding framework for designing and helping understand the results of a prospective behaviour change implementation strategy employing A&F in Kenyan clinical settings

## Design and methodology

### Study design

The study will have a standard interrupted time-series study (ITS) design with an internal control to evaluate the comparative effectiveness of basic versus enhanced A&F after the introduction of enhanced A&F. The study design will also incorporate a parallel group controlled ITS design to compare the standard enhanced A&F package with a further extended A&F package. A process evaluation will be used to track implementation of both. Facilities will be randomised with an allocation ratio of 1:1 to receive the enhanced and extended pharmacist-delivered A&F intervention with the data-dependent components (package 1), or the enhanced pharmacist-delivered A&F intervention with standard components (package 2), which are explained further below. Participating hospitals and the clerks responsible for collecting de-identified data will be blinded to the initial group assignment, but the researchers administering the interventions and assessing the outcomes will not.

### Study setting

The study will be conducted in partnership with 20 first-referral level hospitals in Kenya purposefully selected to be of at least moderate size and representative of different malaria transmission zones (Table 2). This will involve the patient population admitted to the newborn unit (NBU), a separate unit with a specific clinical and nursing team, where the average age on admission is 0- or 1-day old; most admitted neonates are inborn [39]. These hospitals joined the Clinical Information Network (CIN), a learning health system in Kenya at different calendar time points between 2014 and 2020 [11, 13, 39]. The hospitals receive 3 monthly clinical audit and feedback reports on the quality of care they provide for common conditions, which include a summary of prescription error rates for gentamicin and penicillin [30]. Neonatal team leaders (neonatologists, paediatricians, and nurses) met face to face once or twice annually until 2020 (before the COVID-19 pandemic) to discuss these reports and how to improve clinical care. The pharmacists in these hospitals have not previously been involved in CIN feedback activities except in some hospitals linked to the “Supportive care and antibiotics for severe pneumonia among hospitalized children (SEARCH)” trial [42] where their role is to support correct use of study drugs used on the paediatric wards.

**Table 2:** CIN hospitals newborn units’ characteristics

Indicator	H1	H2	H3	H4	H5	H6	H7	H8	H9	H10	H11	H12	H13	H14	H15	H16	H17	H18	H19	H20
Deliveries per year^a^	6387	4441	6228	4581	5515	2945	9939	2578	6744	8641	11404	5571	5131	21608	8872	3653	2963	4264	13104	2032
Number of still births (%)^a^	180 (3)	195 (4)	172 (3)	150 (3)	203 (4)	42 (1)	213 (2)	47 (2)	191 (3)	231 (3)	237 (2)	169 (3)	87 (2)	521 (2)	196 (2)	105 (3)	130	171	315	32
Admissions^b^	1247	671	1759	905	1524	1038	2644	412	1000	2580	2384	864	1391	4837	2964	427	174	125	1318	221
Outborns^b^ (%)	359 (28.79)	229 (34.13)	36 (2.05)	245 (27.07)	34 (2.23)	0 (0)	88 (3.33)	5 (1.21)	255 (25.5)	29 (1.12)	58 (2.43)	23 (2.66)	206 (14.81)	216 (4.47)	679 (22.91)	64 (14.99)	0 (0)	45 (36)	195 (14.8)	1 (0.45)
Number of medical officers (MOs) dedicated to NBU^c^	0.5	0.5	0.5	1	1.5	0.5	0.5	0.5	0.5	1	0	0.5	1	0.5	0	5	1	1	1	1
Number of paediatricians dedicated to NBU^c^	0.5	0.5	1	0.5	1	0.5	1	0.5	0.5	1	1	1	0.5	0.5	1	6	1	1	1	3
Nurse per day shift^d^	2	1	1	5	6	2	3	3	3	5	4	2	3	2	3	5	2	1	2	1
Nurse per night shift^d^	1	1	2	2	3	2	2	1	1	3	3	1	2	1	2	3	1	1	2	1
Cots in NBU	17	2	41	23	40	17	39	1	10	53	15	4	32	0	60	50	6	13	32	4
Babies share cots	Yes	Yes	No	No	Yes	Yes	Yes	No	Yes	No	Yes	Yes	No	No	No	Yes	Yes	Yes	Yes	No
Incubators^e^	10	2	8	10	7	6	8	3	4	7	8	11	6	6	11	7	6	24	13	2
Babies share incubators	Yes	Yes	Yes	No	Yes	Yes	Yes	Yes	Yes	No	Yes	Yes	No	Yes	Yes	Yes	Yes	No	Yes	No
Birthweight (grams) below which stable LBW are admitted in NBU	2100	2000	2000	2000	2000	2000	2000	1800	1800	2000	2000	1800	1800	1800	2000	1800	1700	2000	1800	2400

^a^Deliveries and still births per year (percentage still births) — Jan 2019–Dec 2019. Source — District Health Information System

^b^All NBU admissions (inborn and outborn neonates) and % of outborn neonates in NBU per year — Jan 2019–Dec 2019, Source CIN-Neonatal Database

^c^MOs/paediatricians dedicated to NBU — fraction time spent in NBU, 0.5 of person implies that the staff works 50% time of 8 am–5 pm working days in the NBU. In 50% of the working period — the staff is in the other paediatric wards

^d^Nurses — includes neonatal nurses (NN) in 7 hospitals (H4, H11, H14, and H15 had one NN each, H7 and H11 had 2 NNs, and H16 had 3 NNs)

^e^Functional equipment as per March 2020

### Outcomes

**Fig. 2:**
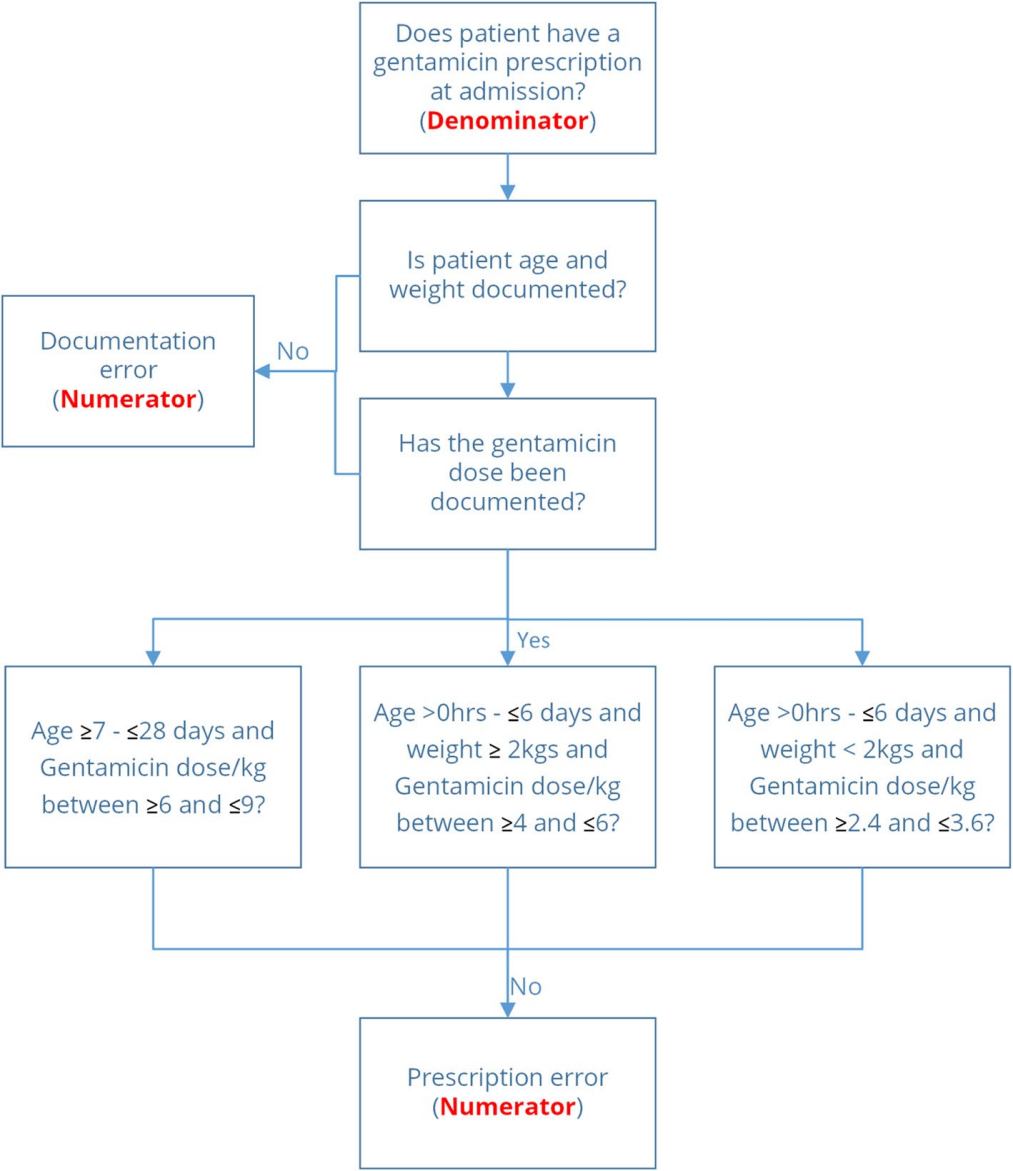
Study primary outcome from patients admitted to the NBUs. Dosage calculations per kilogram allow for ±20% deviation, outside which they are considered errors

The primary outcome of this study is the proportion of patients in newborn units receiving an inaccurate gentamicin prescription. The calculation of the correct prescription according to the Kenyan guidelines is age and weight dependent as illustrated in Fig. 2. Gentamicin prescription is reported in milligram units. From the CP-FIT model, this outcome represents the standard of clinical performance against which clinical behaviour would be measured explicitly (*goal setting*) [20].

For the planned analysis, this study’s target process outcome is at least a 35% reduction in prescription errors from individual hospital baseline error rates with this target based on published evidence of 35–50% reductions in prescription errors in neonatal care from before to after studies [19].

### Intervention

All hospitals already receive regular standardised quarterly A&F reports. The quarterly report is shared via email and as a printed copy to the paediatrician and the hospital manager. The email report sent belongs to a hospital team whose members include the data clerks, hospital records officers, the hospital managers, and the clinical team (i.e. the nurses, medical officers, paediatricians) working in the NBU. The quarterly reports, which are described elsewhere [30], include summaries of the quality of the processes of care, e.g. recording of birth weight and gestational age, and whether or not basic investigations are done and their results documented [30]. The quarterly report also already includes feedback on the correctness of dosing of commonly prescribed medications (e.g. gentamicin and penicillin).

**Fig. 3:**
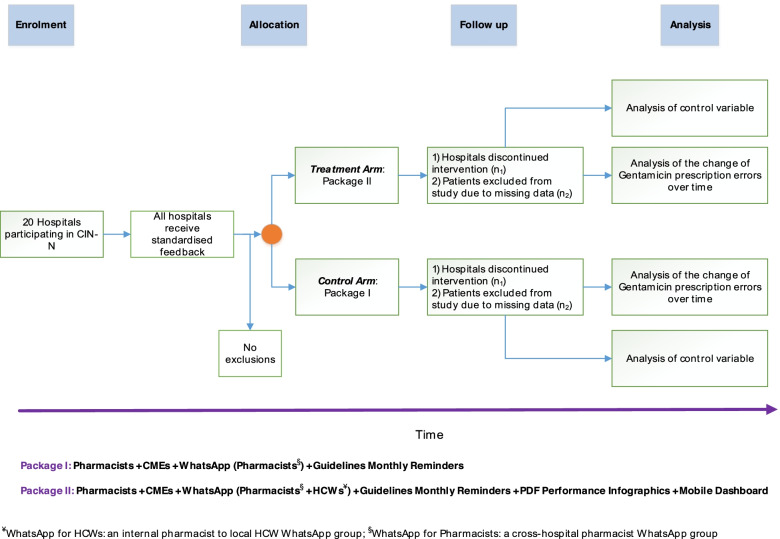
Flow chart of the intervention rollout. The ITS starts prior to random allocation of hospitals

Therefore, there is a baseline A&F intervention already in use in all sites that may be allocated to package 1 or package 2 of the enhanced A&F intervention. If participating hospitals agree, they will be randomised to receive either A&F intervention package 1 or package 2. These packages and their differences are illustrated in Table 3 and Fig. 3. Hospitals receiving package 1 act as the contemporaneous control group for those receiving package 2 to address objective 2. Data from both package 1 and 2 hospitals will be used to address objective 1 of evaluating the comparative effectiveness of enhanced A&F relative to the basic baseline A&F. We will use a control outcome within the ITS design to examine whether improvements in performance (correct prescribing) are more pronounced than improvements that may be linked to underlying secular trends [43]. Such a control outcome should not be affected by the intervention but would be affected by confounding events [43]. The planned control outcome is incorrect penicillin prescription in the same patient population, since 99% of neonates with gentamicin prescription also have a penicillin prescription. In contrast to gentamicin, other first-line antibiotics are considerably less likely to cause patient harm if there are prescription errors (e.g. penicillin), or from reported evidence, typically have lower prescription error rate, or are uncommon in LMICs routine hospital settings like Kenya, thereby giving gentamicin a higher priority in terms of need to intervene [8, 29, 34]. Feedback on both gentamicin and penicillin errors is still being provided throughout this study by the CIN quarterly reports, which will continue throughout the study. Specific ITS analyses will compare effects of package 2 over 1 in the parallel control group study design component.

**Table 3:** Proposed A&F intervention components

#	Intervention component	CP-FIT hypotheses^a^: feedback interventions are more effective when as follows:	Package 1	Package 2	Control mechanism
1	The pharmacists have proposed roles as QI champions/facilitators. They will be supported to conduct a preliminary session for orientating clinical interns into the study when they start their 3-month rotation in paediatric and newborn wards. They will also encourage the nursing staff to identify prescription dosing errors and politely feed this back to the medical staff together with the paediatricians. They will help disseminate monthly reminders on dosing instructions during their physical interactions with NBU ward staff	a) *Champions*: Supportive individuals in the organisation dedicated to making changes a success b) *Competing priorities*: Clinical teams have minimal additional responsibilities and/or competing priorities c) *Workflow fit*: Feedback and action fit alongside existing organisational and team ways of working These elements contribute to effectiveness by promoting credibility of the feedback, limiting the resources needed to provide or act on feedback, and employing social influence in support of a need for behaviour change	☒	☒	Control variable
2	The pharmacists will also conduct 2-monthly routine continuous medical education (CME) sessions and review the performance A&F summaries with the newborn unit team for 15 min in the monthly morbidity and mortality meetings for the whole team or any other suitable forum at the local hospital	a) *Knowledge and skills in clinical topic* and *quality improvement*: Feedback targets health professionals with greater capability in the clinical topic under focus b) *Source knowledge and skill*: Delivered by a person or organisation perceived to have an appropriate level of knowledge or skill c) *Delivery to a group*: Feedback delivered to groups of recipients These elements contribute to effectiveness by relying on social influence to enhance feedback credibility and acceptance, building HCWs knowledge and skills to facilitate action, and, when emphasising a common goal, leveraging teamwork to target HCWs’ perception, intention, and behaviour	☒	☒	Control variable
3	The pharmacists will also be members of a WhatsApp group whose purpose is to facilitate conversations about prescription practices between fellow pharmacists in hospitals in the same study arm. The membership of this WhatsApp group is limited to pharmacists only. The WhatsApp group will be used to disseminate monthly reminders on dosing instructions to be shared with the rest of hospital-specific clinical team	a) *Peer discussion*: Feedback encourages recipients to discuss their performance with peers This element targets feedback perception and intention, by leveraging social influence to break down feedback’s complexity, and identifies possible practice improvements	☒	☒	Control variable
4	The pharmacists will also be members of an additional “within hospital” WhatsApp group whose purpose is to facilitate conversations about prescription practices with their hospital’s healthcare workers posted to the NBUs	a) *Delivery to a group*: Feedback is delivered to groups of recipients b) *Peer discussion*: Feedback encourages recipients to discuss their feedback performance with peers c) *Problem-solving and teamwork*: Feedback supports recipients to identify and develop solutions to reasons for suboptimal performance d) *Action planning*: Feedback provides solutions to suboptimal performance (or support recipients to do so) These elements target feedback’s actionability by evaluating if practice context is compatible with the expected target goals. They promote perception and intention by leveraging social influence to break down feedback’s complexity when identifying possible practice improvements.		☒	“Control” arm
5	An interactive digital application platform that is mobile-friendly and auto-updated monthly used to deliver the enhanced A&F report summaries. The content of the interactive A&F feedback platform will be made up of three visualisations^b^	a) *Automation*: Data collection and analysis are (near) automatic b) *Active delivery*: They “push” feedback messages to recipients rather than requiring them to “pull” c) *Usability*: Feedback delivered employs user-friendly designs d) *Performance level, timeliness, trend, and benchmarking*: Feedback uses recent data to communicate when recipients’ current performance has room for improvement, how recipients’ current performance in relation to their past performance, and compares recipients’ current performance to that of other NBUs e) *Importance, controllability, and relevance*: Feedback focuses on meaningful goals perceived to be in HCWs control and relevant to their role These elements seek to improve perception of and interaction with feedback and provide a relative advantage based on whether the *cost* of deploying feedback is considered inexpensive in terms of time, human, or financial resources. They also seek to promote social influence on (and perception of) feedback while solidifying its actionability, credibility, and acceptance and supporting intention for behaviour change		☒	“Control” arm
6	Enhanced A&F soft-copy (PDF) infographic report generated monthly outlining the proportion of patients who received erroneous gentamicin prescriptions, delivered to the NBU team. These additional A&F reports will be delivered to both the hospital pharmacists, the consultant paediatrician or neonatologist in charge of the neonatal unit, senior nurses, and the medical staff working on rotation in the unit for the duration of the study	a) *Usability*: Feedback delivered employs user-friendly designs b) *Performance level, timeliness, trend, and benchmarking*: Feedback uses recent data to communicate when recipients’ current performance has room for improvement, how recipients’ current performance in relation to their past performance, and compares recipients’ current performance to that of other NBUs c) *Importance, controllability, and relevance*: Feedback focuses on meaningful goals perceived to be in HCWs control and relevant to their role These elements seek to improve perception of and interaction with feedback and provide a relative advantage based on whether the *cost* of deploying feedback is considered inexpensive in terms of time, human, or financial resources. They also seek to promote social influence on (and perception of) feedback while solidifying its actionability, credibility, and acceptance and supporting intention for behaviour change		☒	“Control” arm

^a^Concepts are expounded upon in detail elsewhere: (*Brown, B., Gude, W.T., Blakeman, T. et al. Clinical Performance Feedback Intervention Theory (CP-FIT): a new theory for designing, implementing, and evaluating feedback in health care based on a systematic review and meta-synthesis of qualitative research. Implementation Sci 14, 40 (2019).*10.1186/s13012-019-0883-5)

^b^Discussed in-depth in Additional file 1: Supplementary Table 1

There is currently little interaction between clinical teams and pharmacists for medication prescription such as gentamicin which are reconstituted and administered in the ward; pharmacists tend to come in when potentially toxic medications such as chemotherapy are administered and have oversight of outpatient department prescriptions. However, using CP-FIT to inform the design of the enhanced A&F intervention encouraged us to consider the potentially pivotal role pharmacists might play, as theorised by CP-FIT, by providing enhanced A&F interventions to the usual inpatient care team.

Details of the feedback components of the interactive digital application platform that is mobile-friendly and auto-updated monthly and used to deliver the enhanced A&F report summaries, together with the proposed PDF infographic, are provided in Additional file 1: Supplementary Table 1 and Supplementary Figs. 1–3.

The feedback visualisations are limited to three to reflect HCWs finite capacity to handle feedback [20]. They aim to minimise complexity while automating active delivery and matching resources available to HCWs; we believe they target relevant elements required to influence clinical behaviour change [20]. Because HCWs have strong beliefs regarding how care should be provided which in turn influences their interactions with feedback, the provision of a WhatsApp avenue is to help facilitate and evaluate if the perceptions, acceptance, and intentions teased from their interaction with the A&F intervention align with the observed clinical behaviours [20]. The WhatsApp group will also include two research paediatricians to encourage discussions and raise questions.

#### Installation and access to the mobile-based dashboard

A member of the research team will upload the dashboard Android application (i.e. app) onto the Google Play store. Anonymised patient data from the CIN hospitals randomised to package II intervention which are already being backed up by KWTRP will be used to generate aggregate summaries to allow the dashboard to be populated; the dashboard is populated by aggregate summary data only. HCWs in the CIN hospitals randomised to package II intervention will be able to access the hospital-specific gentamicin prescription safety dashboard through their smartphone devices using their individual-specific, site-linked login credentials. The login credentials follow industry standard OAuth 2.0 authentication and security framework to mitigate against unauthorised application access; only authorised users can access the remotely populated dashboard [44]. The user and hospital data are not stored within the Android app minimising the risk of data breach if the application is hacked. However, to minimise risk of the Android app being hacked, software engineering techniques of obfuscating the app have been employed [45]. The dashboard smartphone application will be accessible throughout the study duration. The delivery of the dashboard on a mobile platform is meant to facilitate easier access to the A&F performance summaries. Distribution of the dashboard application will be publicised to the participating HCWs in package II facilities through the local WhatsApp groups mentioned in the Intervention section and during physical CME seminar meetings organised by the pharmacists or CIN research staff. Package I hospital clinicians will not be provided with similar login credentials.

### Eligibility criteria

Referral-level hospitals with (1) high-quality gentamicin prescription baseline clinical data for neonatal inpatient care and (2) a standardised A&F routine clinical improvement cycle to track prescribing practices in neonatal inpatient care are eligible for this study. To our knowledge, only facilities that have historically been involved with the CIN (Table 2) satisfy this requirement.

De-identified data from all patients admitted to the selected hospitals’ NBUs who are under the age of 28 days (i.e. neonates) who receive gentamicin drug prescription on admission are eligible for inclusion into the analysis regardless of gestational age as in Kenya prescriptions are based on weight and postnatal age rather than gestation at birth. Only the patient population with full data on age, weight, and the dose of the prescription will be included in the outcome measurement since these data are needed to measure errors. Numbers of admissions where prescribing information is inadequate to calculate dose accuracy (e.g. missing weight) will also be measured and reported. Inadequate gentamicin prescription documentation currently stands at < 1% of all prescriptions documented within CIN in the last 24 months.

In addition to pharmacists participating from all hospitals’ all HCWs rotated into (or posted in), the NBUs of the hospitals receiving the package 2 intervention are eligible for inclusion into the aspect of the study that explores comments in the pharmacists and local hospital WhatsApp group discussions respectively after due informed consent processes. Click-stream data (defined as a detailed log of how participants navigate through the Android application when using it, which typically includes the within-app pages visited, time spent on each within-app page, how they arrived on the within-app page, and where they went next) from mobile dashboard activity of the HCWs receiving enhanced A&F package 2 will also be used in additional analysis with informed consent.

### Randomisation

We will apply restricted randomisation to achieve greater equivalence between total population size across arms and the baseline prescription error rate [46] and allocate half of the participating CIN hospitals to package 1 with the remainder assigned to package 2 (Fig. 2). This study’s application of restricted randomisation will ensure that facilities have similar characteristics based on the number of neonates receiving gentamicin prescription and the historical levels of facility-based gentamicin prescription errors from CIN data. Randomisation will also increase the likelihood that facility-based characteristics are balanced across the control and experimental study arms by minimising selection bias. During the intervention period and the analysis stage, the participating hospitals and clerks capturing prescribing data will be blinded to the random allocation process, but the research staff will not. Sequence generation for random allocation will use the *anticlust* package in R [47, 48]. Table 4 illustrates the expected randomisation and is generated from the most recent pre-intervention data.

**Table 4:** Difference in outcome event rate across the study arms in the latest 3 months (before introduction of enhanced A&F)

Study arm^**a**^	Patients with incorrect gentamicin prescription (***n***)	All patients with a gentamicin prescription (***n***)	Rate^**b**^	95% ***CI***^**b**^
Package 1	221	1569	0.141	0.125–0.159
Package 2	218	1566	0.139	0.123–0.157
Pooled	439	3135	0.140	0.128–0.153

^a^Hospitals assigned using restricted randomisation to ensure balanced event rate

^b^The arms are not significantly different, statistically

### Data collection procedures and management

Methods of collection and cleaning of data in the CIN are reported in detail elsewhere [30, 49]. In summary, clinical data for neonatal admissions to the hospitals within the CIN are captured through structured neonatal admission record (NAR) forms coupled with standard treatment sheets that are approved by the Ministry of Health. The NAR prompts the clinician with a checklist of fields including patient biodata, clinical assessment, admission and discharge diagnoses, and record of outcome (survival or death). The CIN supports one data clerk in each hospital to extract data from paper medical records, nursing charts, treatment charts, and available laboratory reports each day after a newborn’s discharge into the primary data collection tool developed in Research Electronic Data Capture (REDCap). Automated error checking happens at the point of entry by daily review, every week centrally, and both are complemented by regular external data quality assurance reviews [49]. A minimal dataset — which is unsuitable for our planned analyses — is collected for (1) admissions during major holiday breaks, (2) admissions when the data clerk was on leave, and (3) on a random selection of records in hospitals where the workload is very high. This process is explained in detail elsewhere [49, 50].

All data will be stored in secure KEMRI-Wellcome Trust Research Programme servers with specified researchers provided password-protected access. Data held on these servers are backed up in mirror servers also within the KEMRI-Wellcome Trust Research Programme. Data on quality of care provided to the KEMRI-Wellcome Trust as part of this collaborative proposal are made available in de-identified form derived from medical records. The primary data are therefore owned by the hospitals and their counties with the Ministry of Health. Research staff will not have permission to share the data without further written approval from both the KEMRI-Wellcome Trust Data Governance Committee and the Facility, County, or Ministry of Health as appropriate to the data request.

### Data analysis and statistical methods

This study involves all healthcare workers assigned to work in the NBUs of the participating hospitals. On average, there is one paediatrician, one medical officer, 3-day nurses, and 2-night nurses in a typical NBU unit in this study (Table 2). The number of clinicians working in a specific NBU varies over time based on hospital-specific staff rotation routines, county health system hiring practices, and whether medical training institutions are in session or not. The number of interns (both nurses and medical officers) remains difficult to assess.

#### Interrupted time series (ITS) sample size

There are two quantitatively testable hypotheses. We have adopted a sample size calculation approach that uses generalised estimating equations (GEE) specified in a form that is suited for testing both hypotheses (Additional file 2). Given the complex study design, the GEE approach specified in Additional file 2 has been used to estimate study power of the controlled ITS analysis using a simulation technique that arises naturally from the underlying data model and typically assumed by power and sample size equations. Our approach is applicable to our count outcome and ITS design, and it easily accommodates complex design features such as different and multiple treatment interventions and different site-specific cluster effects [51]. Our analyses will apply the treated analysis principle: all data from patients fitting the inclusion criteria who receive an evaluable admission gentamicin prescription will be analysed. Data on gentamicin prescription error rates in CIN hospitals from end of 2020 to end of 2021 will be used as pre-intervention period data and from the following 12 months after the intervention introduction (i.e. from mid-2022) as the post-intervention data.

Inpatient admissions and data collection during the COVID-19 pandemic period were largely unaffected [52] and will therefore include these admissions in the study, but data collected during major health workforce labour strikes will be omitted. Summary statistics with discontinuity analysis will be reported for the omitted strike period data. For the ITS analysis, based on the levels of erroneous prescribing across practices in the CIN neonatal study sites, we assume a baseline risk of 14% at the pooled CIN level (pooled rate from Table 4), with the intervention posited to reduce it to 9.1% (i.e. 35% reduction). From the GEE specified in detail in Additional file 2, we estimate that with 690 patients per month across the 20 CIN hospitals, our study will have 90% power to detect this 35% reduction of prescription error with a statistical significance of 0.05. Sample size analysis at the individual hospital level revealed that 19/20 of the hospitals did not have sufficient patient numbers per month with gentamicin prescription to facilitate separate within hospital time-series analysis. Currently, the average CIN admissions to NBUs with a gentamicin prescription at admission that is likely to be eligible are 1123 patients per month (with a standard deviation of 47 admissions across the CIN hospitals).

#### Interrupted time series analysis

We will apply a segmented linear mixed effects model with an autoregressive covariance structure on the proposed interrupted time deries (ITS) design, specified as a “natural experiment” that accounts for the pre-intervention trends in the study outcomes [53]. Comparison of pre-intervention to post-intervention trends of the study outcomes addresses this study’s first objective (i.e. *evaluating if enhancing A&F intervention approaches over and above existing use of feedback reports reduces the prevalence of gentamicin prescribing errors in neonatal inpatient hospital care over time*). Informed by previous findings [19], our hypothesised impact model assumes both immediate (level) and month-to-month (slope) changes following the implementation of the intervention. We anticipate observing a slope plus level change (i.e. changes in the trend in the study outcome) of between 35 and 50% reduction in gentamicin prescription errors in neonatal care based on published evidence [19]. Given our negative binomial modelling approach (Additional file 2), the outcome for objective 1 will be reported as an incidence rate ratio.

For the second objective (i.e. *To evaluate if more intense relative to less intense theory-informed A&F is effective in reducing gentamicin prescribing errors in inpatient neonatal care*), the ITS regression model used to address the first research question also includes a binary covariate term for comparing the differences in study outcome trend due to the study packages, with the less-intense package as the reference category. The significance test of the coefficient for the binary covariate term linked to whether the hospital received the enhanced A&F package will serve as the hypothesis test. We provide further explanation of our analysis approach in methodological supplements in Additional file 2. Given our negative binomial modelling approach (Additional file 2), the outcome for objective 2 will also be reported as an incidence rate ratio.

#### Additional supplementary analyses as part of the process evaluation

As an assessment of fidelity to the study design and intervention rollout, we will also embed a simple process evaluation to check whether CMEs happened, how frequently the HCWs accessed the mobile dashboard, and the pattern of the WhatsApp messages volume after sharing of the A&F summary reports. A research team member will observe some CME meetings with the aim to visit at least 3 hospitals in each arm, identified as 2 performing less well and one performing well, and take field notes of the way in which CMEs are conducted and discussions that take place during CMEs. No audio recording will be conducted during CMEs, only note-taking by the research team.

These CME observations may be curtailed by future COVID-19 pandemic lockdown measures. While every effort will be made to collect this data in person and in line with the KEMRI and government’s guidance on site visits, where in-person data collection is not possible, 1–2 research team members will join the CMEs virtually, through either video or voice calls to observe and follow along with the CME session while taking notes. A debrief session with the pharmacist will be held before and after the virtual observations.

Click-stream data (defined as a detailed log of how participants navigate through the Android application during when using it, which typically includes the within-app pages visited, time spent on each within-app page, how they arrived on the within-app page, and where they went next) from mobile dashboard activity of the HCWs receiving enhanced A&F package II will also be analysed by the research team. This data will contain the name of the page interface, the time the HCW accessed the within-app page, the amount of time the HCW spent on the page, and the page that the HCW navigated to next. The click-stream data is limited to the intervention Android app activity.

Using messages shared on the study’s pharmacists WhatsApp group (expected to have between 8 and 12 clinicians/nurses per hospital over the 12 months), we will explore group members’ participation as the intervention progresses at the end of the study period. The messages shared will be collated, de-identified, and thematically analysed according to their source, target, timing, and content. The analysis of the shared messages on WhatsApp will explore the reception, comprehension, and acceptance of the feedback by the HCWs (*Interaction*, *Perception*, and *Acceptance*, respectively) and planned behavioural responses that may be attributed to the feedback (*Intention* and *Behaviour*) and any barriers to behaviour change [20]. The use of CP-FIT will serve as a starting point for these analyses, but we will be open to identifying issues that are not captured in CP-FIT.

#### Analysis software

The analyses for objectives 1 and 2 will be conducted using R software version 4.0.2 [47] and the *NBZIMM* [54] library*.* The thematic text analysis of WhatsApp messages will be done using Python software version 3.8 [55] together with *NetworkX* [56] and *NLTK* [57] libraries.

### Time frame/duration of the project

Subject to obtaining scientific and ethics approval, we presume that the study’s first face, which involves baseline data collection 12 months prior to intervention introduction, will wrap up at the end of April 2022. The intervention phase will run 12 months afterwards, with integrated analysis and report writing running concurrently. This study has been designed to be conducted mostly remotely. We do not expect any interruption in data collection and abstraction in case the country goes into lockdown again due to the ongoing COVID-19 pandemic; routine patient data collection from the CIN — the data platform for this study — has remained largely unaffected by previous rounds of lockdowns [52]. The WhatsApp and CMEs interventional components are largely unaffected by lockdown measures and therefore require little or no contingency planning.

### Ethics approvals

The analyses described in this protocol have been approved by the KEMRI’s Scientific and Ethical Review Committee (SERU #4378) and the Oxford Tropical Research Ethics Committee (OXTREC #574-21). Any future study protocol modifications require pre-approval from these committees. All facilities/individuals that agree to take part in efforts to improve neonatal care will be free to withdraw their collaboration at any time with no penalty. Individual patient consent for the de-identified data on gentamicin doses will not be required. However, informed consent from HCWs to collate and analyse their WhatsApp and mobile-dashboard click-stream data will be sought by research team (Additional file 3: Study Tools 3 a, b, and d). Participating clinicians can withdraw at any point. The results of this analysis will be shared with the Kenyan Ministry of Health and will also be submitted to peer-review publications and for presentation at international conferences.

## Discussion

The work proposed engages directly to improve patient care, and thus, we will be applying the results as part of the study (in the form of feedback) and its efforts to improve care on NBU. Emerging results will be shared with the counties and the MoH and key concerns highlighted as part of efforts to ensure high quality, safe care is provided in Kenyan hospitals. The findings of the current study will also be used in the development of the guidelines and policy formulation governing the use of gentamicin.

We also hope the work will develop better scalable and effective quality improvement approaches, better information systems, and improve health workers’ motivation to focus on improved neonatal outcomes. In the past work, improved tools have been adopted and implemented nationally by the national- and county-level ministries of health, and we will work with ministries, the Kenya Paediatric Association, and hospitals to promote sustained use of improved tools after the project.

All of this should enable health workers to deliver more accurate drug prescribing during clinical care. We hope the better practices will be spread by the professional associations and by formal authorities such as MoH in Kenya, while wider lessons may influence NBU care across the region.

## Supplementary Information

**Additional file 1: Supplementary Table 1.** Feedback components in the interactive mobile-based dashboard and PDF infographics. **Supplementary Figure 1.** Score card reporting deviation from explicit targets. **Supplementary Figure 2.** Peer comparison of Gentamicin prescription error by patient sub-groups. **Supplementary Figure 3.** Hospital-specific performance trends by age-groups [11, 13, 20, 30, 58, 59].

**Additional file 2.** Methodological supplements [53, 60–63].

**Additional file 3.** Informed Consent Forms and Tools.

## Data Availability

The datasets generated and/or analysed during the current study are not publicly available due to the primary data being owned by the hospitals and their counties with the Ministry of Health. The research staff do have permission to share the data without further written approval from both the KEMRI-Wellcome Trust Data Governance Committee and the Facility, County, or Ministry of Health as appropriate to the data request. Requests for access to primary data from qualitative research by people other than the investigators will be submitted to the KEMRI-Wellcome Trust Research Programme data governance committee as a first step through dgc@kemri-wellcome.org, who will advise on the need for additional ethical review by the KEMRI Research Ethics Committee.

## Abbreviations

A&F: Audit and feedback
CIN: Clinical Information Network
CME: Continuous medical education
CP-FIT: Clinical Performance Feedback Intervention Theory
EHRs: Electronic health records
HICs: High-income countries
HCWs: Healthcare workers
ITS: Interrupted time series
KPA: Kenya Paediatric Association
LMICs: Low- and middle-income countries
MoH: Ministry of Health
NBU: Newborn unit
QI: Quality improvement
SERU: KEMRI’s Scientific and Ethics Review Unit
SSA: Sub-Saharan Africa
WHO: World Health Organization

## References

[1] Donaldson LJ (2017). Medication without harm: who’s third global patient safety challenge. Lancet.

[2] European Medicines Agency (2015). Medication errors.

[3] Panagioti M (2019). Prevalence, severity, and nature of preventable patient harm across medical care settings: systematic review and meta-analysis. BMJ.

[4] Mekonnen AB (2018). Adverse drug events and medication errors in African hospitals: a systematic review. Drugs Real World Outcomes.

[5] Okello N (2020). Antibiotic prescription practices among prescribers for children under five at public health centers III and IV in Mbarara district. PLoS One.

[6] Baraki Z (2018). Medication administration error and contributing factors among pediatric inpatient in public hospitals of Tigray, northern Ethiopia. BMC Pediatr.

[7] Alghamdi AA (2019). Prevalence and nature of medication errors and preventable adverse drug events in paediatric and neonatal intensive care settings: a systematic review. Drug Saf.

[8] Eslami K (2019). Identifying medication errors in neonatal intensive care units: a two-center study. BMC Pediatr.

[9] IGME, U. Levels and trends in child mortality. New York: United Nations Inter-agency Group for Child Mortality Estimation (UN IGME); 2017.

[10] Mohsin-Shaikh S (2019). The impact of electronic prescribing systems on healthcare professionals’ working practices in the hospital setting: a systematic review and narrative synthesis. BMC Health Serv Res.

[11] English M (2020). Programme theory and linked intervention strategy for large-scale change to improve hospital care in a low and middle-income country-a study pre-protocol. Wellcome Open Res.

[12] Leis JA, Shojania KG (2017). A primer on PDSA: executing plan–do–study–act cycles in practice, not just in name. BMJ Qual Saf.

[13] Irimu G (2018). Approaching quality improvement at scale: a learning health system approach in Kenya. Arch Dis Child.

[14] Gachau S (2017). Does audit and feedback improve the adoption of recommended practices? Evidence from a longitudinal observational study of an emerging clinical network in Kenya. BMJ Glob Health.

[15] Ayieko P (2019). Effect of enhancing audit and feedback on uptake of childhood pneumonia treatment policy in hospitals that are part of a clinical network: a cluster randomized trial. Implement Sci.

[16] Avery AJ (2012). A pharmacist-led information technology intervention for medication errors (PINCER): a multicentre, cluster randomised, controlled trial and cost-effectiveness analysis. Lancet.

[17] Tuti T (2017). A systematic review of electronic audit and feedback: intervention effectiveness and use of behaviour change theory. Implement Sci.

[18] Nzinga J, McGivern G, English M (2018). Examining clinical leadership in Kenyan public hospitals through the distributed leadership lens. Health Policy Plann.

[19] Nguyen M-NR, Mosel C, Grzeskowiak LE (2018). Interventions to reduce medication errors in neonatal care: a systematic review. Ther Adv Drug Saf.

[20] Brown B (2019). Clinical Performance Feedback Intervention Theory (CP-FIT): a new theory for designing, implementing, and evaluating feedback in health care based on a systematic review and meta-synthesis of qualitative research. Implement Sci.

[21] Grimshaw J (2019). Reinvigorating stagnant science: implementation laboratories and a meta-laboratory to efficiently advance the science of audit and feedback. BMJ Qual Saf.

[22] Ivers NM (2014). No more ‘business as usual’with audit and feedback interventions: towards an agenda for a reinvigorated intervention. Implement Sci.

[23] Ivers N, et al. Audit and feedback: effects on professional practice and healthcare outcomes. Cochrane Database Syst Rev. 2012;2012(6):CD000259. 10.1002/14651858.CD000259.pub3. 10.1002/14651858.CD000259.pub3 PMC11338587 22696318

[24] UNICEF. Levels and trends in child mortality 2020. UNICEF; 2021. p. 18. https://www.unicef.org/reports/levels-and-trends-child-mortality-report-2020. Retrieved on January, 2020.

[25] Downie L (2013). Community-acquired neonatal and infant sepsis in developing countries: efficacy of WHO’s currently recommended antibiotics—systematic review and meta-analysis. Arch Dis Child.

[26] Organization, W.H (2011). WHO model list of essential medicines: 17th list, March 2011.

[27] World Health Organisation (2017). WHO recommendations on newborn health.

[28] Okomo U (2019). Aetiology of invasive bacterial infection and antimicrobial resistance in neonates in sub-Saharan Africa: a systematic review and meta-analysis in line with the STROBE-NI reporting guidelines. Lancet Infect Dis.

[29] Musiime GM (2015). Risk of gentamicin toxicity in neonates treated for possible severe bacterial infection in low-and middle-income countries: systematic review. Trop Med Int Health.

[30] Maina M, et al. Using a common data platform to facilitate audit and feedback on the quality of hospital care provided to sick newborns in Kenya. BMJ Glob Health. 2018;3(5):e001027. https://gh.bmj.com/content/3/5/e001027, 10.1136/bmjgh-2018-001027. 10.1136/bmjgh-2018-001027 PMC6150140 30258654

[31] Maina M (2020). Antibiotic use in Kenyan public hospitals: prevalence, appropriateness and link to guideline availability. Int J Infect Dis.

[32] Aluvaala J (2015). Assessment of neonatal care in clinical training facilities in Kenya. Arch Dis Child.

[33] English M (2004). A randomised, controlled trial of once daily and multi-dose daily gentamicin in young Kenyan infants. Arch Dis Child.

[34] Fuchs A (2018). Reviewing the WHO guidelines for antibiotic use for sepsis in neonates and children. Paediatr Int Child Health.

[35] Muinga N (2020). Digital health systems in Kenyan public hospitals: a mixed-methods survey. BMC Med Inform Decis Mak.

[36] Ogero M (2020). Examining which clinicians provide admission hospital care in a high mortality setting and their adherence to guidelines: an observational study in 13 hospitals. Arch Dis Child.

[37] English M (2020). The paediatrician workforce and its role in addressing neonatal, child and adolescent healthcare in Kenya. Arch Dis Childh.

[38] McKnight J (2019). Evaluating hospital performance in antibiotic stewardship to guide action at national and local levels in a lower-middle income setting. Glob Health Action.

[39] Irimu G (2021). Neonatal mortality in Kenyan hospitals: a multisite, retrospective, cohort study. BMJ Glob Health.

[40] Peek N (2020). Evaluation of a pharmacist-led actionable audit and feedback intervention for improving medication safety in UK primary care: an interrupted time series analysis. PLoS Med.

[41] Ivers NM, Barrett J (2018). Using report cards and dashboards to drive quality improvement: lessons learnt and lessons still to learn. BMJ Qual Saf..

[42] National Institutes of Health (2019). A study to compare different antibiotics and different modes of fluid treatment for children with severe pneumonia (SEARCH).

[43] Lopez Bernal J, Cummins S, Gasparrini A (2018). The use of controls in interrupted time series studies of public health interventions. Int J Epidemiol.

[44] Hardt D (2012). The OAuth 2.0 authorization framework.

[45] Balachandran V, Tan DJ, Thing VL (2016). Control flow obfuscation for android applications. Comput Secur.

[46] Sedgwick P. Restricted randomisation. BMJ. 2012:344:e1324. https://www.bmj.com/content/344/bmj.e1324, 10.1136/bmj.e1324.

[47] R Core Team (2020). R: a language and environment for statistical computing.

[48] Papenberg M, Klau GW. Using anticlustering to partition data sets into equivalent parts. Psychol Methods. 2020. 10.1037/met0000301. 10.1037/met0000301 32567870

[49] Tuti T (2016). Innovating to enhance clinical data management using non-commercial and open source solutions across a multi-center network supporting inpatient pediatric care and research in Kenya. J Am Med Inform Assoc.

[50] Tuti T (2016). Improving documentation of clinical care within a Clinical Information Network: an essential initial step in efforts to understand and improve care in Kenyan hospitals. BMJ Glob Health.

[51] Arnold BF (2011). Simulation methods to estimate design power: an overview for applied research. BMC Med Res Methodol.

[52] Wambua S (2021). The indirect impact of COVID-19 pandemic on inpatient admissions in 204 Kenyan hospitals: an interrupted time series analysis. PLoS Glob Public Health.

[53] Mascha EJ, Sessler DI (2019). Segmented regression and difference-in-difference methods: assessing the impact of systemic changes in health care. Anesth Analg.

[54] Zhang X (2018). Negative binomial mixed models for analyzing longitudinal microbiome data. Front Microbiol.

[55] Van Rossum G (2007). Python programming language. USENIX annual technical conference.

[56] Hagberg A, Swart P, Chult DS (2008). Exploring network structure, dynamics, and function using NetworkX.

[57] Loper E, Bird S (2002). Nltk: the natural language toolkit. arXiv preprint cs/0205028.

[58] Gude WT (2019). Clinical performance comparators in audit and feedback: a review of theory and evidence. Implement Sci.

[59] Lahitani AR, Permanasari AE, Setiawan NA (2016). Cosine similarity to determine similarity measure: study case in online essay assessment. 2016 4th international conference on cyber and IT service management.

[60] Stevens S (2016). Analysing indicators of performance, satisfaction, or safety using empirical logit transformation. BMJ.

[61] Linden A (2015). Conducting interrupted time-series analysis for single-and multiple-group comparisons. Stata J.

[62] Bernal JL, Cummins S, Gasparrini A (2017). Interrupted time series regression for the evaluation of public health interventions: a tutorial. Int J Epidemiol.

[63] Irimu G (2018). Tackling health professionals’ strikes: an essential part of health system strengthening in Kenya. BMJ Glob Health.

