# Supplementary material for: Evaluation of an audit and feedback intervention to reduce gentamicin prescription errors in newborn treatment (ReGENT) in neonatal inpatient care in Kenya: a controlled interrupted time series study protocol
Source: Implement Sci. 2022 May 16;17:32. doi: 10.1186/s13012-022-01203-w (PMC9109356; doi:10.1186/s13012-022-01203-w)
Supplement: Supplementary file 1 — Additional file 1: Supplementary Table 1. Feedback components in the interactive mobile-based dashboard and PDF infographics. Supplementary Figure 1. Score card reporting deviation from explicit targets. Supplementary Figure 2. Peer comparison of Gentamicin prescription error by patient sub-groups. Supplementary Figure 3. Hospital-specific performance trends by age-groups [11, 13, 20, 30, 58, 59]. [file 13012_2022_1203_MOESM1_ESM.docx]

**1. Addendum figures and tables**

| Supplementary Table 1: Feedback components in the interactive mobile-based dashboard and PDF infographics | | |
| --- | --- | --- |
| Visualisation | Feedback Strategy | Reasoning with an illustrative CP-FIT hypothesis being targeted* |
| Supplementary Figure 1-3 | Emphasise explicit targets (i.e., Goal target), explicit behavioural target of <1% prescription error rate.  **Feature in figure:**  *Supplementary Figure 1: Uses text to clearly state the target level of error in prescribing being targeted.*  *Supplementary Figure 2, 3: Uses a horizontal red dashed line to demarcate the level above which the error was considered problematic.* | Explicit targets are posited to facilitate action by reducing complexity of the feedback, making it easier for recipients to know what constitutes ‘good performance’ and therefore what requires a corrective response [59].  This goal represents high quality professional care and is guided by the collaborative expert opinion from the ongoing neonatal learning health system [12, 14, 30].  Support NBU team’s discussion of potential causes of the errors and come up with solutions to affect the three points under “improve” in Supplementary Figure 3 e.g., during CMEs; Posited to facilitate problem-solving negotiation between changes at individual patient level versus system/ organisational changes  From CP-FIT, feedback is hypothesised to be more effective when feedback variables include goal importance, relevance, and controllability. This rely on acceptance and intention feedback cycle processes whose explanatory mechanism is based on actionability, compatibility and relative advantage the feedback provides. |
| Supplementary Figure 2 | Emphasise benchmarking against identifiable individual peers (which are a subset of other hospitals deemed comparable by the hospital).  Peer comparison illustrated where performance reported includes multiple comparisons to hospital-selected peers^¥^.  **Feature in figure:**  *Supplementary Figure 2: Uses bar-charts to compare hospital performance to peers* | This approach to benchmarking is posited to increase effectiveness when recipients choose the most relevant peers for comparison and increases their sense of competition knowing that their own performance is also known to other “peer” hospitals^ǂ^, applying a social influence mechanism [1, 59].  From CP-FIT, feedback is hypothesised to be more effective when data collection and analysis methods highlight benchmarking together with performance level. This strategy relies on perception, intention, and behaviour feedback cycle processes whose explanatory mechanism is based on social influence in addition to feedback’s actionability, compatibility with current practice, and complexity. |
| Supplementary Figure 2-3 | Provide patient-lists linked benchmarks within the A&F summary visualisation. This strategy is achieved by breaking down the nature of the errors by patient sub-groups in line with the constitution of the primary outcome that communicate the relative importance of the feedback contents.  **Feature in figure:**  *Supplementary Figure 2, 3: Reporting performance categorised by patient sub-groups which vary by a combination of age and/or weight and requiring differing levels of prescription.* | This strategy is posited to facilitate feedback acceptance by increasing credibility through helping recipients assess variation between patient groups and judge whether potential discrepancies are clinically significant, providing specific information that can help prioritise needed actions [59]. Feedback interventions that successfully and directly support clinical behaviours for individual patients and more targeted patient-groups are most effective [1].  From CP-FIT, feedback is hypothesised to be more effective when feedback display shows patient lists used to generate performance level while communicating where clinical appropriateness for patients can be further optimised. This component(s) rely on verification, acceptance, perception, intention, and behaviour feedback cycle processes whose explanatory mechanism is based on credibility and complexity in addition to actionability, and compatibility the feedback provides. |
| Supplementary Figure 3 | Provide performance trends  **Feature in figure:**  *Supplementary Figure 3: Line plots over time showing recipients’ current performance in relation to their past performance.* | This strategy is posited to decrease A&F complexity and facilitate action by supporting recipients to identify and interpret: (a) If the reference period includes sufficient time points at regular intervals on the performance topic, and, (b) when clinical performance requires action [59]. Strategy is also posited to increase the observability of the potential benefits of feedback intervention and in-turn, induce emotions in HCWs by demonstrating how their clinical performance has changed over time owing to their taken actions.  Strategy facilitates acceptance of feedback by increasing its credibility because performance is measured during a reference period that includes multiple time points (e.g., to eliminate the possibility of one-time coincidentally poor performance) [59].  From CP-FIT, feedback is hypothesised to be more effective when where its implementation increases performance observability and trend. This rely on perception, acceptance and complexity feedback cycle processes whose explanatory mechanism is based on credibility in addition to actionability, compatibility and relative advantage the feedback provides. |
| Note:  ^ǂ^ To achieve this, each hospital will be provided with a list of top three similar “peer” hospitals based on the number of neonates per month receiving gentamicin and top-three performing hospitals. Each hospital’s peers are generated from comparing its performance metrics to other hospitals over calendar time on the size of population prescribed gentamicin using the cosine similarity index [60].  * Not meant to be an exhaustive list. Concepts are expounded upon in detail elsewhere: (*Brown, B., Gude, W.T., Blakeman, T. et al. Clinical Performance Feedback Intervention Theory (CP-FIT): a new theory for designing, implementing, and evaluating feedback in health care based on a systematic review and meta-synthesis of qualitative research. Implementation Sci 14, 40 (2019).* [*https://doi.org/10.1186/s13012-019-0883-5*](https://doi.org/10.1186/s13012-019-0883-5))  ^¥^ The decision on whether to compare the hospital’s performance to best performing hospitals or similarly sized hospitals will be left to the pharmacists and therefore allowed to vary from hospital to hospital and will be recorded. | | |


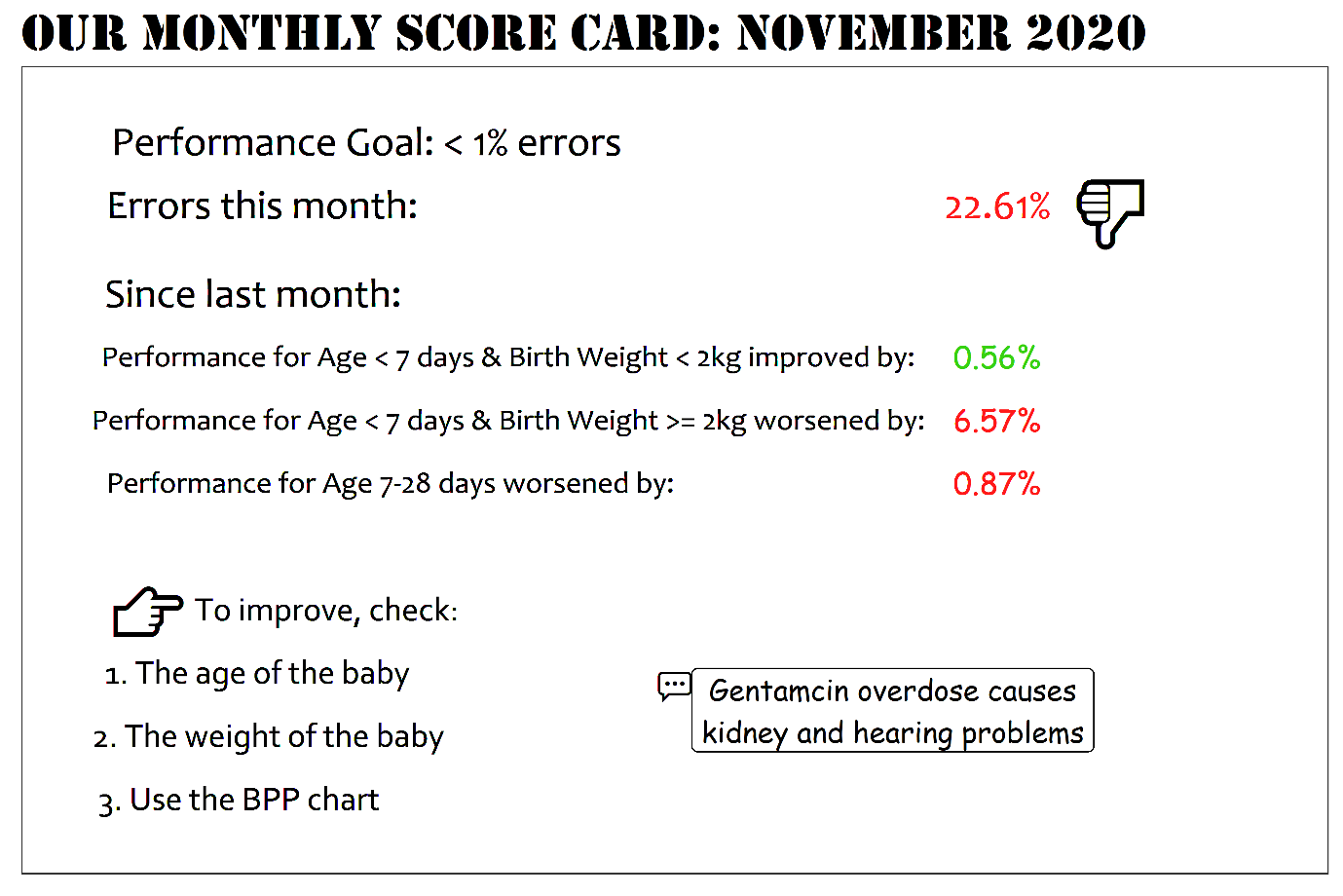


*Supplementary Figure 1: Score card reporting deviation from explicit targets*


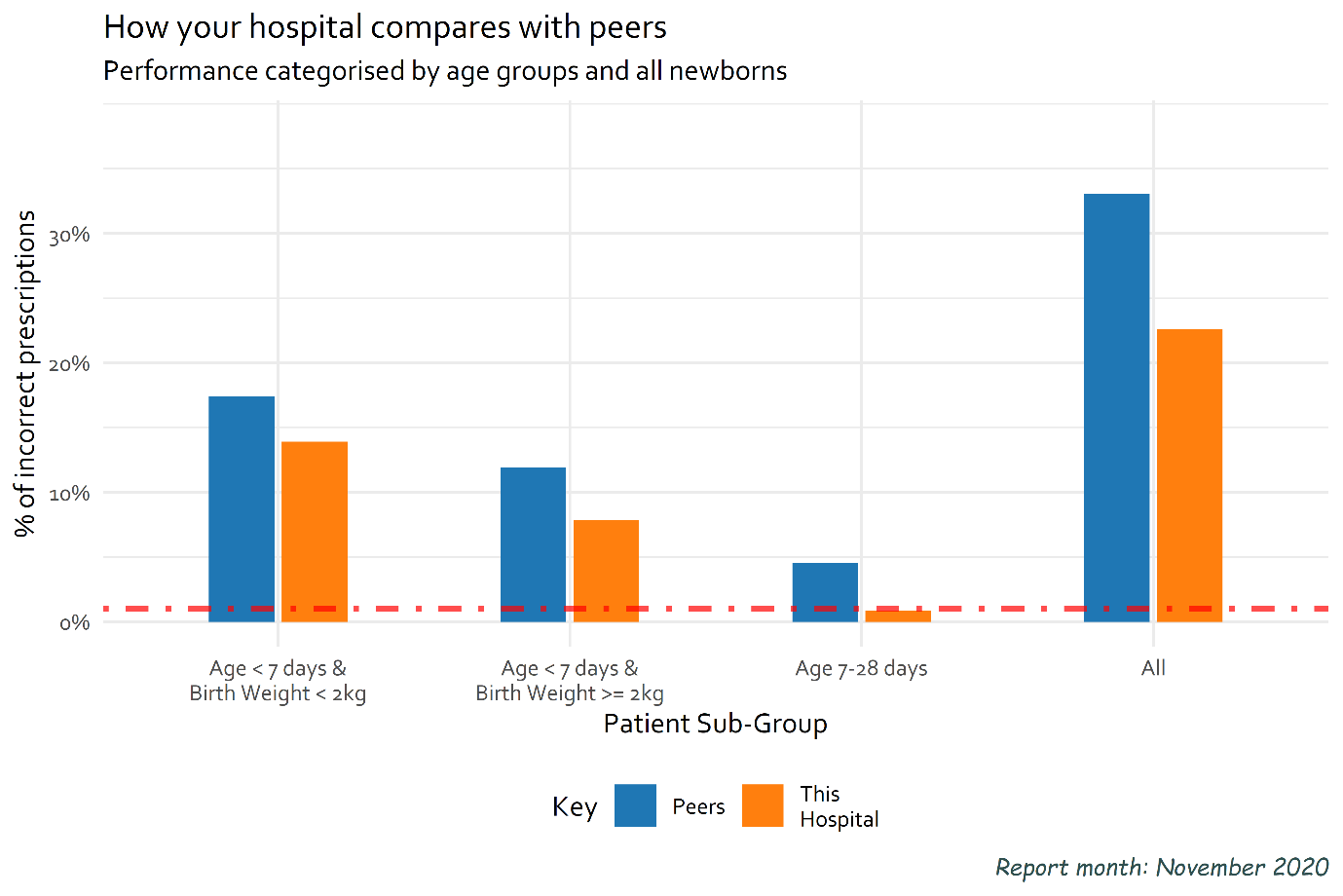


*Supplementary Figure 2: Peer comparison of Gentamicin prescription error by patient sub-groups*


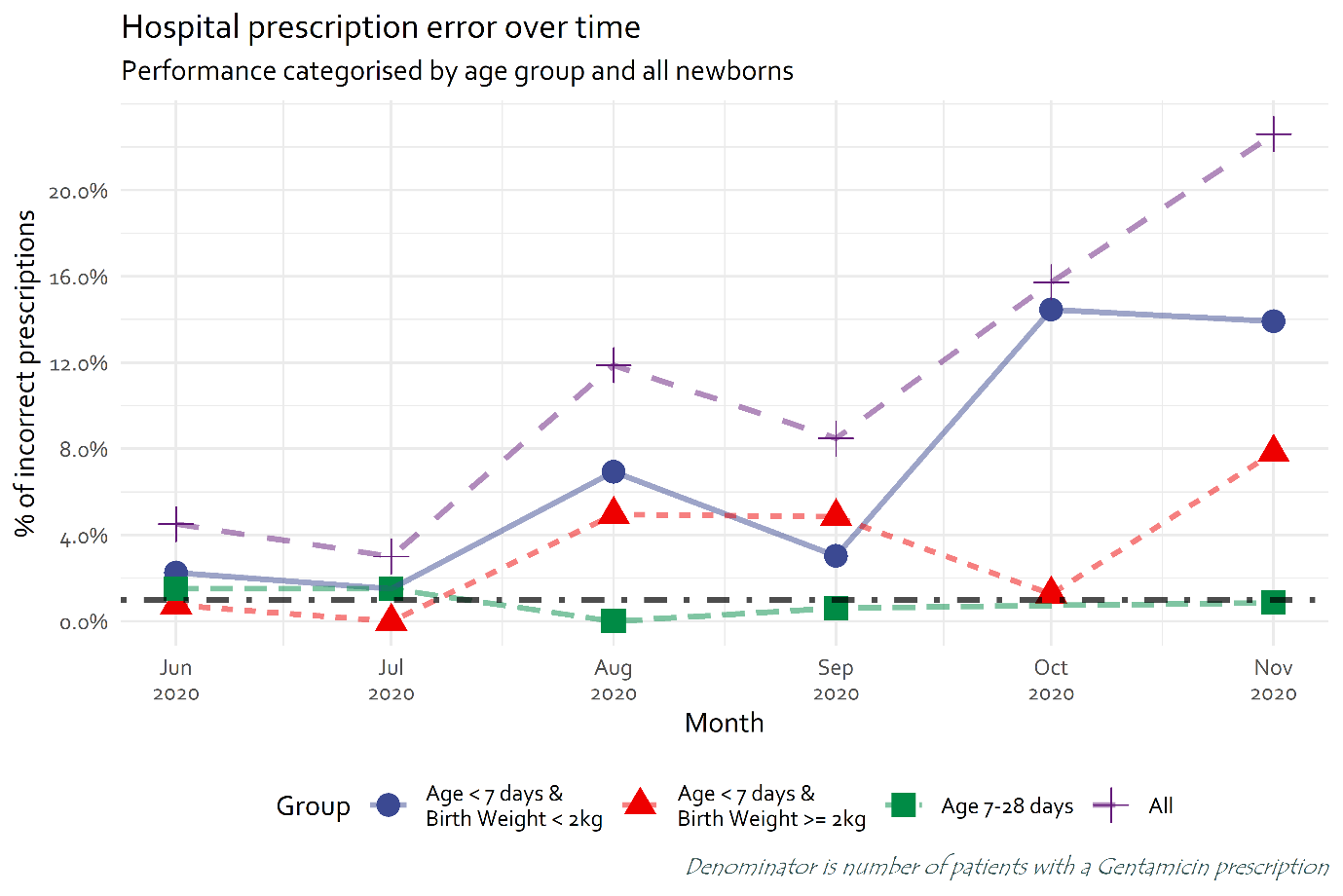


*Supplementary Figure 3: Hospital-specific performance trends by age-groups*
