# Supplementary material for: Evaluation of an audit and feedback intervention to reduce gentamicin prescription errors in newborn treatment (ReGENT) in neonatal inpatient care in Kenya: a controlled interrupted time series study protocol
Source: Implement Sci. 2022 May 16;17:32. doi: 10.1186/s13012-022-01203-w (PMC9109356; doi:10.1186/s13012-022-01203-w)
Supplement: Supplementary file 2 — Additional file 2. Methodological supplements [53, 60–63]. [file 13012_2022_1203_MOESM2_ESM.docx]

**2. Methodological supplements**

Our modelling approach for ITS will assume a negative binomial distribution of the outcome to account for any overdispersion that might exist in the data [53]. The primary outcome will be the count of patients with a prescription error (*dependent variable*) regressed on the offset of the number of patients given gentamicin prescription in that month (*independent variable*) and the time in months (*independent variable*). To help account for and quantify hospital variability, we will include a random intercept term at the hospital level. The log-linear form of the fixed terms of this model is illustrated by Equation 1.

$$log\left( \mu_{t} \right)= \beta_{0}+\beta_{1}T_{t}+\beta_{2}X_{t}+ \beta_{3}\left( T_{t}-t_{0} \right)X_{t}+ \beta_{4}X_{con\_var}+ \beta_{5}\left( T_{t}-t_{0} \right)X_{int\_arm}+g\left( \Gamma_{t-1};\theta\right) + e_{t} (1)$$

Where:

$\Gamma$ _𝑡−1_ = {𝑌_0_, ..., 𝑌_𝑡−1_, 𝜇_0_, ..., 𝜇_𝑡−1_}, 𝜇_𝑡_ = 𝐸(𝑌_𝑡_|$\Gamma$ _𝑡−1_), is the mean of 𝑌_𝑡_ conditioning on the past responses and means, the function 𝑔 joints current outcome with past outcomes that are correlated in the time series i.e. the autocorrelation structure.

𝑇_𝑡_ is the actual time of the study,

𝑡_0_ is the time point of the intervention,

𝑋_𝑡_ is the binary indicator for the second phase of the study i.e., when the improvement strategy was implemented

𝑋*_con_var_* is the count indicator for the number of patients per month who satisfy the control variable in the study

𝑋*_int_arm_* is the binary indicator for whether the facility is in the enhanced A&F study arm (Package II) of the study

𝛽_0_ is the regression intercept representing the starting level of the log of the conditional mean,

𝛽_1_ is the slope of the log of the conditional mean before the implementation of the improvement strategy,

𝛽_2_ is the change in the level of the log of the conditional mean caused by the improvement strategy introduced versus the baseline A&F strategy.

𝛽_3_ is the difference in the slopes of the log of the conditional mean caused by the improvement strategy introduced versus the baseline A&F strategy.

𝛽_4_ is the difference in the level of the log of the conditional mean associated with the enhanced A&F strategy introduced relative to the change in the control variable in the NBUs.

𝛽_5_ is the difference in the slopes of the log of the conditional mean associated with the difference in the enhanced A&F strategy package II relative to the less enhanced A&F package I in the NBUs.

For an arbitrary two‐sided statistical test with the null hypothesis 𝐻_0_∶ 𝛽 = 0 versus the alternative 𝐻_1_∶ 𝛽 ≠ 0, where 𝛽 represents the regression coefficients from equation 1, we will test five null hypotheses:

1. 𝛽_2_ = 𝛽_3_ = 0, to test whether any changes (level, trend or both) exist after the intervention.
2. 𝛽_2_ = 0, to test the change on level after intervention packages introduction; and
3. 𝛽_3_ = 0, to test any trend changes after intervention packages introduction.
4. 𝛽_4_ = 0, to test any level changes after intervention packages introduction in the control variable.
5. 𝛽_5_ = 0, to test any slope changes after intervention introduction due to package II intervention relative to package I.

The modelling specification approach we plan to use, including the recasting of the outcome, avoids the limitations of using linear models for modelling trends in improvement (i.e. treating the primary outcome as a percentage) being subject to ceiling and floor effects [61]; It also allows for the model’s link function to convert the model terms into a linear form, as expected by ARIMA models [61, 62].

For the ITS analysis, to ensure that we adequately reflect the correlations between the repeated outcome measurements of each hospital which decrease with time lag (i.e. autocorrelation), the regression model will include a term for an autocorrelation structure of order one [62]. While the planned statistical analysis for intervention effectiveness will be done after 12 months, we also plan to assess intermediate changes in the outcome after 4 months, and then every other month afterwards.

***Methodological issues and sensitivity analyses***

Given that we are applying an autoregressive covariance structure in our mixed effects model, this will minimise any bias in reported findings that might arise from autocorrelation (i.e. consecutive observations being similar to one another compared to those that are further apart) [63]. In the proposed analysis, we will also explore and report whether there might be any time-varying confounders that might affect the primary outcome [63]; For example, In the Kenyan context, we could anticipate encountering the problem of health system labour strikes which occur frequently [64]. In the event of these strikes happening, we will conduct a change-point analysis to examine the strike’s effects on patient admissions. If influential, we will discuss the implications on findings and whether they could safely be excluded from the data for the corresponding strike periods.

Since we have adopted the negative binomial modelling approach together with the use of an offset term in the ITS model, this will minimise any bias in findings that might arise from overdispersion. The offset term serves to reflect the change in the denominator from month to month since the underlying patient populations sizes will most likely differ for each of the observed counts at the hospital level even if the rate is similar. Additionally, in case of non-linear trends in the outcome, Fourier terms will be added to the models to address the non-linear trends, and likelihood ratio tests conducted to examine significance of the Fourier terms [63].
