# Supplementary material for: Evaluation of an audit and feedback intervention to reduce gentamicin prescription errors in newborn treatment (ReGENT) in neonatal inpatient care in Kenya: a controlled interrupted time series study protocol
Source: Implement Sci. 2022 May 16;17:32. doi: 10.1186/s13012-022-01203-w (PMC9109356; doi:10.1186/s13012-022-01203-w)
Supplement: Supplementary file 3 — Additional file 3. Informed Consent Forms and Tools. [file 13012_2022_1203_MOESM3_ESM.docx]

**3. Informed Consent Forms and Tools**

**
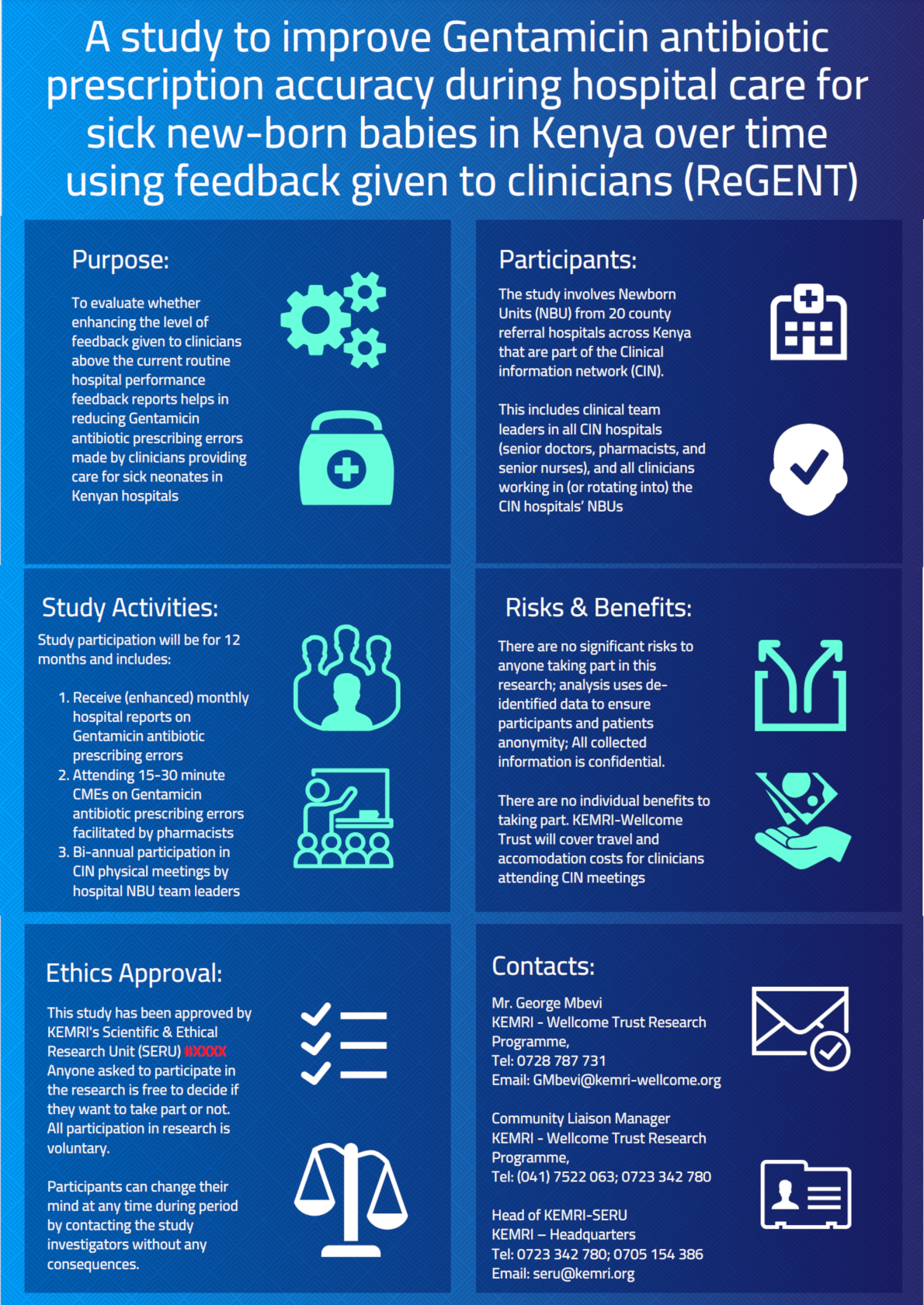
Study Form 3a - Information poster for use on noticeboards to create awareness on ReGENT study in clinicians working in New-Born Unit (NBU) of CIN hospitals**

**Study Form 3b - Information sheet and consent form for hospital pharmacists (adapted for either a CME seminar observation or individual participation in WhatsApp discussions)**

**Study title:** Evaluation of an audit and feedback intervention trial to Reduce Gentamicin Prescription Errors in Newborn Treatment (ReGENT) in neonatal inpatient care in Kenya: A controlled interrupted time series study

**Lay Title:** A study to improve Gentamicin antibiotic prescription accuracy during hospital care for sick new-born babies in Kenya over time using feedback given to clinicians

| Institution | Investigators |
| --- | --- |
| KEMRI Wellcome Trust Research Programme | Prof. Mike English, Dr Timothy Tuti, Dr Jalemba Aluvaala, Dr Michuki Maina, Prof Grace Irimu, George Mbevi, Livingstone Mumelo*,* John Wainaina, Daisy Chelangat, Kefa Wairoto, Dolphine Mochache |
| London School of Hygiene and Tropical Medicine | Dr Lucas Malla |
| Nuffield Department of Medicine, University of Oxford, UK | Ms. Christiane Hagel |
| Division of Population Health, University of Manchester, UK | Dr. Benjamin Brown |

You are being asked to take part in a study. The box below tells you important things you should think about before deciding to join the study. We will provide more detailed information below the box. Please ask questions about any of the information before you decide to participate. You may also wish to talk to others (for example, your line manager, family, or friends) about this study, before agreeing to join.

| **Key Information for You to Consider** |
| --- |
| - **Voluntary Consent**. You are being asked to volunteer for a research study. You can choose whether you would like to participate or not. If you do agree, you can change your mind at any time and withdraw from the research. This will not affect you now or in the future. - **Purpose**. We want to learn more about how to help hospitals provide better care for the sick babies that need to stay in the hospital for treatment soon after they are born. We are doing this by working with at least 20 hospitals that we call the ‘network’. We will work with the pharmacists, doctors and nurses from the network hospitals to find better ways of working that improve prescribing quality and safety. We will learn how to measure some aspects of babies’ care and progress and use this information to promote improvements in care. We will also be looking at how we can improve teamworking between doctors, nurses and other staff and help make care safer. - **Duration.** The phase of this study which you are being asked to participate in, will last for 12 months. - **Procedures and Activities.** We will ask you to take part in between 4 to 6 Continuous Medical Education (CMEs) seminars throughout the year, be involved in WhatsApp discussions on how to improve prescription practices at the hospital, and optionally, track your newborn unit’s prescription error performance through a mobile platform. - **Risks or disadvantages.** There are no known harms in this study that could happen to you if you join. In this study, there is a possibility that you might be a little bit inconvenienced by the time it takes to participate in the prescribing-based pharmacist-led CMEs. The researchers aim to minimise this inconvenience by encouraging the pharmacists to limit the length of the CMEs to around 15-30 minutes. - **Benefits**. There are no direct benefits in this study. However, the outputs from this study will contribute to the improvement of hospital care for newborns. If the audit and feedback interventions are successful, the strategies that we are testing could be adopted much more widely across Kenya. |

**Who is carrying out this study and what is this study about?**

This study is being carried out by Kenya Medical Research Institute (KEMRI). KEMRI is a government organization that carries out medical research to find better ways of preventing and treating illness in the future for everybody’s benefit and is working with the University of Nairobi and the Kenya Paediatric Association to conduct this study.

In this research, we want to learn more about how to help hospitals adopt safer medication prescription practices for the sick babies that need to stay in the hospital for treatment soon after they are born. We are doing this by working with 20 hospitals that we call the ‘network’. We will work with the pharmacists, doctors and nurses from the network hospitals to find better ways of working that improve prescription quality and safety. We will track the outcomes (i.e., Gentamicin antibiotic prescribing error) from the anonymised patient data collected from the network hospitals and use this information to promote improvements in care. We will also be looking at how we can improve teamworking between pharmacists, doctors, nurses, and other staff and help make medication prescribing safer. To evaluate the whether the approach is helping we would like to spend some time in the hospital observing Continuous Medical Education (CME) seminars linked to prescribing practices. We will use WhatsApp to facilitate discussions with health care workers at various levels of seniority and use routine hospital data to trace the prescribing errors on babies admitted to the newborn unit. Due to COVID 19 preventive measures, we may not be able to conduct physical discussion with the healthcare workers. In such cases, we may use online collaboration platforms such as WhatsApp to keep the discussion going.

**Why do you want to observe the WhatsApp discussions and what does it involve?**

- We are interested in the experience of pharmacists/nurses/doctors/hospital managers with an understanding of / experience of medication prescription and provision in newborn units as exploring these experiences can help us understand how things really are and whether efforts to make improvements on prescribing errors are working or not.
- The discussion will take place via an appropriate online platform which is WhatsApp. Health workers who are part of the same clinical team in your newborn unit will be also members of the same WhatsApp group. We would like to observe and collect anonymised data from the WhatsApp group on the reception, comprehension, and acceptance of the feedback and CME seminars by the clinical team, including your contributions, and any planned practice change based on the feedback and seminars.
- If you do not want WhatsApp messages analysed, you can tell us, and we can stop this.

**Are there any risks or disadvantages to me taking part?**

- The Continuous Medical Education (CME) seminars should take approximately 15 to 30 minutes of your time. The WhatsApp discussions and your contributions can be at whatever time you chose, depending on how strongly you feel about the topic being discussed.
- We do not believe there are any risks to your taking part as we aim to ensure the confidentiality of all participants so that no comments can be directly linked to any person.
- In the case of electronic communications in online consent, you should be aware that this form is not being sent from a ‘secure’ http server such as the kind used in to handle credit card transactions. There is therefore a small possibility that responses signed forms could be viewed by unauthorized parties, such as computer hackers. However, the researchers will take appropriate measures to ensure the data received is secured according to the data security policies that have been put in place at the programme.

**Are there any advantages to me of taking part?**

There are no individual benefits to taking part. In talking to us, you will contribute to knowledge on how efforts to improve care on newborn units in Kenyan hospitals can work best. This may help other people in Kenya and elsewhere in the future, for example through making the current approaches to improvement better or developing new strategies to improve care.

**Data Protection**

- KEMRI-Wellcome Trust Research Programme and the University of Oxford are the data controllers with respect to your personal data, and as such will determine how your personal data is used in the study. KEMRI-Wellcome Trust Research Programme and the University of Oxford will process your personal data for the purpose of the research outlined above. Research is a task that is performed in the public interest.

**Who will have access to the information I give?**

- All of our documents are stored securely in locked cabinets and on password protected computers. The knowledge gained from this research will be shared in summary form, without revealing individuals’ identities, with study participants, hospital managers, policy makers and professional regulatory bodies.
- Anonymised data will be stored in a secure KEMRI-Wellcome Trust Research Programme repository and may be used for future research with reasonable requests and approved by KEMRI.
- In future, information collected or generated during this study may be used to support new research by other researchers in Kenya or other countries on improving neonatal service delivery. In all cases, we will only share information with other researchers in ways that do not reveal individual participants’ identities. For example, we will remove such as their names and where they live and replace this information with number codes. Any future research using information from this study must first be approved by a local or national expert committee to make sure that the interests of participants and their communities are protected.
- KEMRI will keep any personally identifiable information about you from this study for 10 years after the study has finished in accordance with applicable Data Protection Requirements both in Kenya and in the UK. You have the right to access the personal data we hold that pertains to you, to object to or make corrections to the processing of all or part of the personal data. However, this might be limited due to coding which might make unblinding difficult.
- In order to do this study, we will share anonymised individual and summary information we collect or generate with our partner institution (University of Oxford) in ways that do not reveal individual participants’ identities.
- University of Oxford is responsible for ensuring that Oxford staff involved in the trial in Kenya adhere to the safe and proper use of any personal information you provide, solely for research purposes. You can contact the research team for any further information about how your data will be managed. Further information about your rights with respect to your personal data is available from <https://compliance.admin.ox.c.uk/individual-rights>

**Who has allowed this research to take place?**

All research at KEMRI has to be approved before it begins by its Scientific and Ethical Review Unit who look carefully at planned work. They must agree that the research is important, relevant to Kenya and follows nationally and internationally agreed research guidelines. This includes ensuring that all participants’ safety and rights are respected.

**What will happen if I refuse to participate?**

All participation in research is voluntary. You are free to decide if you want to take part or not. If you do agree you can change your mind at any time without any consequences.

**What if I have any questions?**

You are free to ask me any question about this research. If you have any further questions about the study, you are free to contact the research team using the contacts below:

George Mbevi (study coordinator) KEMRI Wellcome Trust Research Programme, P.O. Box 43640, Nairobi 00100. Telephone: 0730 162 000

**If you want to ask someone independent anything about this research please contact:**

Community Liaison Manager, KEMRI Wellcome Trust Research Programme, P.O. Box 230, Kilifi. Telephone: 041 7522 063, Mobile 0723 342 780 or 0705 154 386

***And***

The Head, KEMRI Scientific and Ethics Review Unit, P. O. Box 54840-00200, Nairobi; Telephone numbers: 0717 719477; 0776 399979 Email address: [seru@kemri.org](mailto:seru@kemri.org)

**KEMRI-Wellcome Trust Research Programme consent form for** *A study to improve Gentamicin antibiotic prescription accuracy during hospital care for sick new-born babies in Kenya over time using feedback given to clinicians*

I have had the study explained to me. I have understood all that has been read/explained and had my questions answered satisfactorily and **I agree to take part to take part in this research.**

**Please initial the sentences that reflect your choices, and then sign below:**

_____ I do wish to be notified by investigators in the event of research findings of possible importance to my family members or myself. **Yes No**

______ I agree that the study team use the identifier that I have provided (name, telephone number, country ID number, etc.) to locate me in the future. **Yes No**

**I also agree for notes detailing the Continuous Medical Education (CME) seminar to be taken and WhatsApp chat messages for this study being analysed.**

I understand that I can change my mind at any stage and it will not affect me in any way.

| **Signature:** |  | **Date:** |  | |
| --- | --- | --- | --- | --- |
|  |  | | | |
| **Participant name:** |  | **Time:** | |  |
|  | ***(please print name)*** |  | | |

--------------------------------------------------------------------------------------------------------------------------------------

I have followed the study SOP to obtain consent from the participant. S/he apparently understood the nature and the purpose of the study and consents to the participation in the study. S/he has been given opportunity to ask questions which have been answered satisfactorily.

**Designee/investigator’s signature:** ____________________________ **Date** ____________

**Designee/investigator’s name:**  _____________________________**Time** ____________

(Please print name)

**THE PARTICIPANT SHOULD NOW BE GIVEN A SIGNED COPY TO KEEP

***APPENDIX FORM 4D ILLUSTRATES INFORMED CONSENT TO BE PROVIDED VIRTUALLY.

**Study Form 3c - Observation guide for the newborn *Continuous Medical Education* (CME) seminar session linked to prescribing practices**

**Research team member will observe the following:**

1. **The nature of work being done**

- A description of the type of CME seminar that is being observed, who is involved in delivering the seminar, and what the seminar topic is, when it was delivered, approximately how many staff are in attendance.

1. **Team Leadership**

- Observe the champions- what work are they doing? Do they provide solutions to suboptimal performance (or support attendees to do so)?
- Do the leadership/champions provide a CME environment that empowers people to understand their roles? Do they ask for opinions? Are they clear and do they share goals / expectations?
- Do all team members express their thoughts and opinions without fear? (note who is saying what and responding to whom (Junior vs senior staff, within senior staff and within junior staff). How are they communicating/discussing these issues -gauge the tone, facial expressions, pauses etc)?
- What is the tone/mood/ in the CME and between staff?

**3. Team goals/Objectives**

- Is there an articulation of the CME goal? Is it deemed relevant to attendees’ roles? Are the staff working towards shared goals? Do they help each other?
- Does the CME seminar address tasks considered to be meaningful and within the control of the recipients but often do not happen in practice?
- Is everyone reminded of important practices and the needs of each baby? Is the medication prescription of babies discussed and prioritised?
- Are team members from all professions working and communicating together well?

**4. Individual competencies / roles**

- How is feedback performance described and prioritised? What is the target health professionals’ attitude about feedback?
- Do more senior team members help support those who are more junior in having greater capability in the clinical topic under focus?
- Do staff follow official guidelines and take care in using standard job-aides linked to prescription practices?

**5. Team interactions**

- Observe team interactions – how are members interacting during the CME – (talking, facial expressions?).
- Do team members raise concerns about quality or safety of prescribing practices, and are they addressed during the CME?

**6. Solving problems**

- What techniques are used to solve problems that are raised during the CME that would affect patient safety from medication prescription and delivery.
- Is there any form of reflective or shared learning during the CME?

**7 Agency**

- Observe if there are differential power issues between and within staff cadres?

**8 Context**

- Record the resources available for use by health care teams, space, and availability of basic materials, raised during the CME.
- Does the team outline plans to undertake changes involving the wider organisation of care provided rather than just individual patients in response to the CME/feedback?

***FOR VIRTUAL OBSERVATIONS, A DEBRIEF CALL WITH THE FACILITATING PHARMACISTS BEFORE AND AFTER THE CME WILL BE USED TO FILL OUT SOME OF THE REQUIRED DETAILS APART FROM VOICE/VIDEO OBSERVATIONS

**Study Form 3d: Electronic consent (E-consent) within-intervention text for pharmacists, paediatrician, medical and clinical officer, and nurse participants (adapted for mobile performance dashboard)**

Title of study: Evaluation of an audit and feedback intervention trial to Reduce Gentamicin Prescription Errors in Newborn Treatment (ReGENT) in neonatal inpatient care in Kenya: A controlled interrupted time series study

Lay Title: A study to improve Gentamicin antibiotic prescription accuracy during hospital care for sick new-born babies in Kenya over time using feedback given to clinicians

Researcher(s): *KEMRI-Wellcome Trust Research Programme* (**Dr.** **Timothy Tuti, Prof. Mike English, Dr. Jalemba Aluvaala, Dr. Michuki Maina, Prof. Grace Irimu, George Mbevi, Livingstone Mumelo*,* John Wainaina, Daisy Chelangat, Kefa Wairoto, Dolphine Mochache**), *London School of Hygiene and Tropical Medicine* (**Dr. Lucas Malla**)*, University of Oxford - NDM* (**Ms. Christiane Hagel**), *University of Manchester* (**Dr. Benjamin Brown**)

Funders: The Wellcome Trust (**London, UK**)

Aim: To evaluate multiple Audit & Feedback intervention approaches to reduce the prevalence of prescribing errors in neonatal inpatient hospital care over time.

Data: The app has been checked for and successfully deters any security vulnerabilities by being digitally signed with a security certificate and using secured encrypted online private and local (in-phone) storage. While using the mobile-based performance dashboard, click-stream statistics (e.g., which graphs, and buttons are clicked) will be collected from the mobile phones that will be used to access the mobile dashboard, if you consent. To add context to prescription error dashboard usage statistics, additional data on *clinical* c*arder, age, years of experience* and *institution* and associated WhatsApp chat messages will be collected. This research does not collect or store personal identifiers and all data collected in this research are anonymised. KEMRI-Wellcome Trust Research Programme and the University of Oxford are the data controllers with respect to your personal data, and as such will determine how your personal data is used in the study. KEMRI-Wellcome Trust Research Programme and the University of Oxford will process your personal data for the purpose of the research outlined above. Research is a task that is performed in the public interest.

Benefits: There are no individual benefits to taking part although we anticipate that using mobile prescription safety dashboard will increase your ability to avoid prescription errors for patients admitted to newborn unit. Due to constrained resources, this study is unable to provide data bundles to clinicians to facilitate WhatsApp communication or reimbursed for any mobile data costs they incur. Through reviewing your performance and experience improving prescribing practice, you will contribute to knowledge on how best to implement innovative training tools that may help other clinicians in lower income countries access training in the future.

Reviewer(s): KEMRI *Scientific & Ethics Review Unit* (SERU, **Protocol #: 4378**), University of Oxford *Oxford Tropical Research Ethics Committee* (OxTREC, **Application #: 574-21**)

Consent: By clicking once on the “agree” button, you are accepting that (1) data from the dashboard and from the form you complete (if you choose to do this) will be recorded anonymously, (2) that the anonymised data (dashboard usage statistics) shared may be used for purposes of research (analysis, inclusion in policy documents and publications), and (3) anonymised individual and summary information collected can be shared within the collaborating institutions (mentioned in researcher’s section) in ways that do not reveal individual participants’ identities. You can withdraw from the research at any time at any time without any consequences by emailing the contacts listed below.

Voluntary Consent: You are being asked to volunteer for a research study. You can choose whether you would like to participate or not. If you do agree you can change your mind at any time and withdraw from the research. You can withdraw from the research any time without any consequences by emailing the contacts listed below.

Contacts: You are free to ask any question about this research by contacting the research team using the following contacts: George Mbevi, KEMRI-Wellcome Trust Research Programme ([GMbevi@kemri-wellcome.org](mailto:GMbevi@kemri-wellcome.org)), KEMRI Scientific and Ethics Review Unit ([seru@kemri.org](mailto:seru@kemri.org)). If you want to ask someone independent anything about this research please contact:

Community Liaison Manager, KEMRI Wellcome Trust Research Programme, P.O. Box 230, Kilifi. Telephone: 0723 342 780 or 0705 154 386,

*And*

The Head, KEMRI Scientific and Ethics Review Unit, P. O. Box 54840-00200, Nairobi; Telephone numbers: 0717 719477; 0776 399979; Email: [seru@kemri.org](mailto:seru@kemri.org)

More info: <https://nest360.org/evidence-based-care/>

<https://wellcomeopenresearch.org/articles/5-265>

**THE PARTICIPANT WILL RECEIVE A DIGITALLY SIGNED PDF COPY TO THEIR WHATSAPP NUMBER TO KEEP
